# Investigating the Theranostic Potential of Elementally Matched [^43^Sc]Sc-PSMA-617 and [^47^Sc]Sc-PSMA-617

**DOI:** 10.1021/acs.molpharmaceut.5c01023

**Published:** 2026-02-18

**Authors:** Shelbie J. Cingoranelli, Emily Putnam, Hailey A. Houson, Grayson R. Gimblet, Sharon Samuel, Volkan Tekin, Suzanne E. Lapi

**Affiliations:** † Department of Radiology, 9968 University of Alabama at Birmingham, Birmingham, Alabama 35233, United States; ‡ Department of Chemistry, University of Alabama at Birmingham, Birmingham, Alabama 35233, United States

**Keywords:** Theranostics, radioscandium, PSMA, Sc-43, Sc-47, targeted radiotherapy, PET

## Abstract

The theranostic approach, which employs diagnostic radiopharmaceuticals to select patients who would benefit from targeted radiotherapy agents, has become an invaluable strategy for effective medical care. Scandium radionuclides offer the advantage of forming elementally matched and chemically identical diagnostic and therapeutic compounds, making them ideal candidates for this strategy. PSMA-617 is an established prostate-specific membrane antigen targeting agent and can be used as a proof of concept to investigate ^43^Sc, the diagnostic nuclide, and ^47^Sc, the therapeutic nuclide, as a theranostic pair. Methods: Cellular uptake, competitive binding assays, and internalization studies were carried out using LNCaP or PC-3 cell lines. [^43^Sc]­Sc-PSMA-617 was used in PET imaging studies in LNCaP or PC-3 tumor models, with time points ranging from 1–9 h. LNCaP tumor-bearing mice injected with [^47^Sc]­Sc-PSMA-617 were imaged using SPECT up to 48 h. A longitudinal study was carried out using LNCaP tumor-bearing mice imaged with [^43^Sc]­Sc-PSMA-617 prior to receiving a therapeutic dose of [^47^Sc]­Sc-PSMA-617. Results: ^43^Sc and ^47^Sc were incorporated into PSMA-617 at radiochemical yields of >99%. Cellular uptake studies demonstrated high uptake and specificity to PSMA receptors for [^47^Sc]­Sc-PSMA-617. *In vivo* PET studies showed specificity of [^43^Sc]­Sc-PSMA-617 while SPECT studies demonstrated tumor retention of [^47^Sc]­Sc-PSMA-617 up to 48 h. [^47^Sc]­Sc-PSMA-617 demonstrated therapeutic efficacy by delaying tumor growth and increasing survival rates from a single administered dose in xenograft models. More importantly, the PET results from [^43^Sc]­Sc-PSMA-617 PET were highly correlated with the therapeutic response from [^47^Sc]­Sc-PSMA-617, showing that ^43^Sc PET data can predict therapeutic outcomes in individual animals from ^47^Sc agents, even in animals sharing a genetic background and implanted with tumors from the same cell line. Conclusions: Two chemically identical, PSMA-targeting radioscandium pharmaceuticals demonstrated *in vivo* stability, specificity and retention in PSMA+ tumor models. A theranostic study showed that a higher ^43^Sc PET SUV_mean_ was strongly correlated to therapeutic response from the ^47^Sc agent, demonstrating that ^43^Sc and ^47^Sc can be used as an elementally matched theranostic pair.

## Graphical abstract

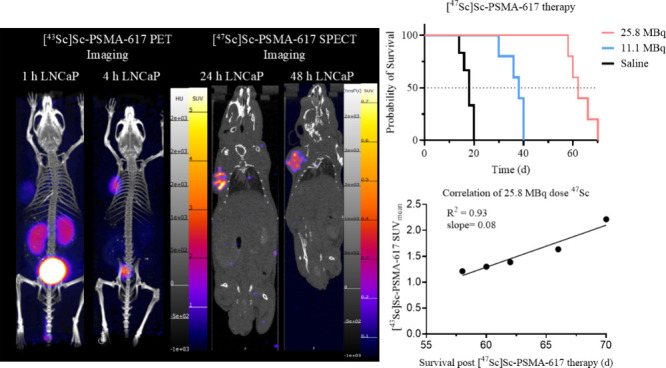

## Introduction

The theranostic landscape continues to expand with the addition of both new targeting moieties and novel radionuclides.^1–8^ Selecting suitable radionuclides for theranostic pairs is based on both the decay emissions of these nuclides and chemical properties. One of the radionuclides should have a decay emission suitable for nuclear imaging, such as a photon with energy suitable for Single Photon Emission Computed Tomography (SPECT) or a positron for Positron Emission Tomography (PET). In contrast, the therapeutic radionuclide should emit alpha (α) particles, beta (β-) particles, Auger electrons, or a combination of these. These diagnostic compounds can be used to select patients who would benefit from the corresponding targeted therapeutic radiopharmaceuticals.

A simplified diagram of the theranostic approach is illustrated in Figure 1. A suitable theranostic pair would enable the synthesis of two chemically similar compounds suitable for imaging and therapy, sharing the same targeting moieties and exhibiting modest differences in their pharmacokinetics.

**Figure 1:**
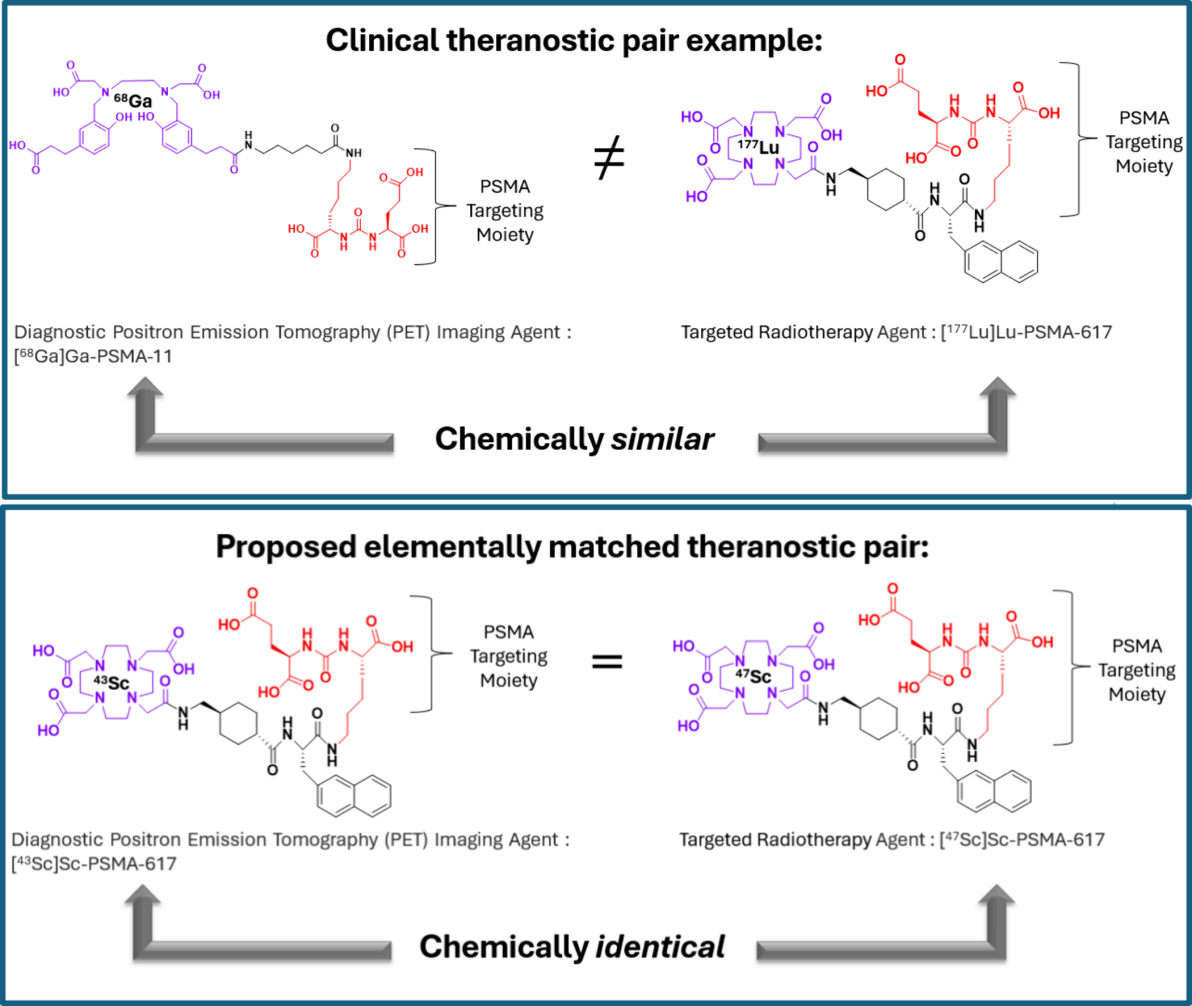
Two examples of theranostic pairs: the clinically used [^68^Ga]­Ga-PSMA-11 with [^177^Lu]­Lu-PSMA-617 and the proposed elementally matched pair [^43^Sc]­Sc-PSMA-617 with [^47^Sc]­Sc-PSMA-617.

Yet, literature studies have shown that there can be differences in the pharmacokinetics of compounds which differ only in the chelated radiometal.^9–13^ Houson et al. reported differences in the pharmacokinetics between [^64^Cu]­Cu-NOTA-NT-20.3, [^55^Co]­Co-NOTA-NT-20.3 and [^68^Ga]­Ga-NOTA-NT-20.3, particularly in the liver uptake at 24 h.^11^ Wei et al. reported differences between [^68^Ga]­Ga-NOTA-hu19 V3 and [^64^Cu]­Cu-NOTA-hu19 V3 in the same tumor model, with [^64^Cu]­Cu-NOTA-hu19 V3 showing lower kidney accumulation than [^68^Ga]­Ga-NOTA-hu19 V3 at 1 h.^9^ Two studies compared differences in PSMA-617 labeled with different radiometals, where Meyer et al. showed that [^225^Ac]­Ac-PSMA-617 had significantly higher kidney uptake at 1 h and tumor uptake at 168h than compared to [^177^Lu]­Lu-PSMA-617 while Umbricht et al. showed that [^44^Sc]­Sc-PSMA-617 *in vivo* kinetics were more similar to [^177^Lu]­Lu-PSMA-617 than [^68^Ga]­Ga-PSMA-617.^10,13^ Elementally matched theranostic compounds, as illustrated in Figure 1, have the advantage of forming chemically identical compounds with identical pharmacokinetics. This chemically identical strategy can employ the diagnostic agent to predict the response from the therapeutic agent and to determine the dosimetry of the therapeutic agent accurately. Table 1 provides a list of elementally matched theranostic pairs of interest.

**Table 1:** Elementally Matched Theranostic Pairs

Rare-Earth Metals			Transition Metals		
Radionuclide	*t*_*1/2*_	Use	Radionuclide	*t*_*1/2*_	Use
^43^Sc	3.89 h	PET	^55^Co	17.53 h	PET
^44^Sc	4.04 h	PET	^58m^Co	9.1 h	Therapy
^47^Sc	3.35 d	SPECT/Therapy	^61^Cu	3.39 h	PET
^86^Y	14.74 h	PET	^64^Cu	12.70 h	PET/Therapy
^90^Y	64.05 h	Therapy	^67^Cu	61.83 h	SPECT/Therapy
^149^Tb	4.12 h	PET/Therapy	^197m^Hg	23.8 h	SPECT/Therapy
^152^Tb	17.5 h	PET/Therapy	^197g^Hg	64.14 h	Therapy
^155^Tb	5.32 d	SPECT			
^161^Tb	6.89 d	SPECT/Therapy			

Halogens			Post-transition Metals		
Radionuclide	*t*_*1/2*_	Use	Radionuclide	*t*_*1/2*_	Use
^76^Br	16.2 h	PET	^72^As	26.00 h	PET
^77^Br	57.04 h	Therapy	^76^As	26.24 h	Therapy
^80m^Br	4.42 h	Therapy	^117^Sb	2.80 h	SPECT
^123^I	13.22 h	SPECT	^118^Sb	5.00 h	PET
^124^I	4.17 h	PET	^119^Sb	38.19 h	Therapy
^131^I	8.02 d	SPECT/Therapy	^203^Pb	51.92 h	SPECT
			^212^Pb	10.62 h	Therapy

The triad of radioscandium nuclides, ^43^Sc, ^44^Sc, and ^47^Sc, has been proposed for theranostic applications.^14^ Both ^43^Sc and ^44^Sc decay via positron emission, making them suitable for diagnostic imaging, while ^47^Sc decays by β- emission, which is ideal for targeted radiotherapy. Recent advances in the production of radioscandium nuclides have achieved high radionuclidic and chemical purity, facilitating their incorporation into radiopharmaceuticals with various chelators.^12,15–31^ The incorporation of these radioscandium nuclides into radiopharmaceuticals yields identical scandium complexes; however, a theranostic study combining ^43^Sc/^44^Sc PET imaging with therapeutic results from ^47^Sc still requires further exploration.

The prostate-specific membrane antigen (PSMA) is overexpressed in castration-resistant metastatic prostate cancers.^3^ The FDA-approved radiopharmaceuticals [^68^Ga]­Ga-PSMA-11 and [^177^Lu]­Lu-PSMA-617 are standard-of-care as part of a theranostic strategy for prostate cancer patients, but are chemically *similar* complexes rather than chemically *identical*.^2,3,5,32–38^ As radioscandium has been shown to form highly stable DOTA-containing compounds, illustrated by two clinical trials using [^44^Sc]­Sc-PSMA-617 and [^44^Sc]­Sc-DOTATOC, scandium radionuclides would have an advantage, as both complexes would now be identical, as either [^43^Sc]­Sc-PSMA-617 or [^44^Sc]­Sc-PSMA-617 with [^47^Sc]­Sc-PSMA-617 ^18–20,29,39–42^

In this study, in-house produced ^43^Sc and ^47^Sc were used to radiolabel PSMA-617. The affinity of [^43^Sc]­Sc-PSMA-617 and [^47^Sc]­Sc-PSMA-617 for PSMA was established using *in vitro* and *in vivo* studies. An imaging and therapy study was conducted in a PSMA+ tumor model, where mice were imaged with [^43^Sc]­Sc-PSMA-617 prior to receiving a therapeutic dose of [^47^Sc]­Sc-PSMA-617, and the response to the therapy was measured and correlated to the imaging results, demonstrating that [^43^Sc]­Sc-PSMA-617 can be used to predict the response to [^47^Sc]­Sc-PSMA-617.

## Method and Materials

Details of all materials for radiolabeling methods and for *in vitro* and *in vivo* studies are provided in the Supporting Information. Radioscandium was produced using a TR24 cyclotron (ACSI, Richmond, BC, Canada), as previously reported.^43–45^

## Cell Lines and Tumor Models

All animal studies were conducted under an approved protocol by the University of Alabama at Birmingham Institutional Animal Care and Use Committee (IACUC), under protocol number IACUC-21613. LNCaP cells (ATCC, Manassas, VA) were grown in RPMI media (10% FBS, 0.1% gentamicin) and PC-3 cells (ATCC, Manassas, VA) were grown in DMEM (10% FBS, 0.1% gentamicin). All cells were incubated at 37 °C with 5% CO_2_. Male, athymic nude mice (6 to 8 wk old, The Jackson Laboratory) were xenografted subcutaneously on the left shoulder with 5 × 10^6^ LNCaP cells in a 1:1 mixture of Matrigel or 5 × 10^6^ PC-3 cells in RPMI media. LNCaP tumors grew to 100 – 150 mm^3^ in 4–6 weeks, while PC-3 tumors grew to 150 mm^3^ in 2 weeks.

### *In Vitro* Studies

#### *In Vitro* Stability

For stability studies, [^47^Sc]­Sc-PSMA-617 was incubated in either human or mouse serum up to 14 d.

#### Saturation Binding

LNCaP cells were incubated with 1 mL of media containing [^47^Sc]­Sc-PSMA-617 at concentrations ranging from 0.01 to 100 nM for 2 h at 37 °C. The cells were then washed with PBS and lysed with 0.2 M NaOH, and the lysate was measured using a gamma counter. A bicinchoninic acid assay (BCA) was performed to measure the total protein concentration.

#### Cellular Uptake and Competitive Binding

For competitive binding assays, 1 mL of media containing 1 nM of [^47^Sc]­Sc-PSMA-617 was added to wells containing either LNCaP cells, LNCaP cells with 100 μM 2-PMPA, PC-3 cells, or PC-3 cells with 100 μM 2-PMPA and incubated for 1 h at 37 °C. The cells were then washed with PBS and lysed with 0.2 M NaOH, and the lysate was measured using a gamma counter. A BCA was performed to measure the total protein concentration.

#### Internalization

LNCaP cells were incubated with 1 mL of media containing [^47^Sc]­Sc-PSMA-617 at a concentration of 1 nM for 0.5, 1, 2, 4, 6, 24, 48, and 72h at 37 °C. A 400 μL of 0.1 M cold citric acid was added to each well and incubated at room temperature (RT) for 5 min before removal. After the addition of citric acid, the cells were lysed with 0.2 M NaOH. Both the citric acid solution and lysate were measured separately on a gamma counter. The results were analyzed by calculating the percentage of activity in the lysed portion relative to the total activity, which is the sum of the citric and lysate fractions.

### *In Vivo* Studies

For all *in vivo* studies, the image modality, imaging parameters (dynamic or static, frames, length), imaging times postinjection, biodistribution time, and number of mice per group are provided in Table 2. Imaging protocols, reconstruction protocols, and image analysis information are provided in the Supporting Information.

**Table 2:** Imaging Parameters for all PET and SPECT Studies

Tumor model	Compound	MBq	nmol	Modality	Imaging Parameters	Imaging Start Time	Biodistribution Start Time	*n*
LNCaP (PSMA+)	[^43^Sc]Sc-PSMA-617	2.2	0.3	PET	60 min dynamic with 10 min frames	Immediately	1.5 h	4
LNCaP (PSMA+)	[^43^Sc]Sc-PSMA-617	2.2	0.3	PET	30 min static	1, 2, or 4 h	1.5, 2.5, or 4.5 h	4
Blocked LNCaP (PSMA+)	[^43^Sc]Sc-PSMA-617 + 2-PMPA	2.2	0.3	PET	30 min static	1 h	1.5 h	4
LNCaP (PSMA+)	[^43^Sc]Sc-PSMA-617	1.6	0.2	PET	30 min static	1, 2.5, 4.5, 7, and 9 h	9.5 h	4
PC-3 (PSMA-)	[^43^Sc]Sc-PSMA-617	2.2	0.3	PET	30 min static	1 h	1.5 h	4
LNCaP (PSMA+)	[^68^Ga]Ga-PSMA-617	2.8	0.3	PET	30 min static	1 h	1.5 h	4
LNCaP (PSMA+)	[^47^Sc]Sc-PSMA-617	4.9	2	SPECT	1 h static	4, 24, and 48 h	25 or 49 h	3

Longitudinal imaging and therapy study								
Group Name	Imaging Agent	MBq	nmol	Modality	Imaging Parameters	Therapy Agent	Injected Activity	*n*
High dose	[^43^Sc]Sc-PSMA-617	2.8	0.8	PET	30 min static at 1 h postinjection	[^47^Sc]Sc-PSMA-617	25.9 MBq, 10.8 nmol	5
Low dose	[^43^Sc]Sc-PSMA-617	2.8	0.8	PET	30 min static at 1 h postinjection	[^47^Sc]Sc-PSMA-617	11.1 MBq, 4.6 nmol	5
Control	[^43^Sc]Sc-PSMA-617	2.8	0.8	PET	30 min static at 1 h postinjection	Saline	100 μL	5

### *In Vivo* PET Studies with [^43^Sc]­Sc-PSMA-617

Mice bearing LNCaP tumors were injected with 100 μL of [^43^Sc]­Sc-PSMA-617­(2.2 ± 0.6MBq) and imaged at various time points from 1 to 9 h, while mice bearing PC-3 tumors were imaged at 1 h. Mice were euthanized after imaging, and organs were collected, weighed, and measured on a gamma counter. Table 2 provides *in vivo* imaging study parameters.

### Comparative Biodistribution of [^43^Sc]­Sc-PSMA-617 and [^68^Ga]­Ga-PSMA-617

LNCaP tumor-bearing mice were injected with 0.3 nmol of either [^43^Sc]­Sc-PSMA-617 (2.2 ± 0.6 MBq) or [^68^Ga]­Ga-PSMA-617 (2.8 ± 0.2 MBq) and imaged at 1 h postinjection. Mice were euthanized at 1.5 h postinjection, organs were collected, weighed and measured on gamma counter.

### *In Vivo* SPECT Imaging with [^47^Sc]­Sc-PSMA-617

LNCaP tumor-bearing mice were administered 2 nmol of [^47^Sc]­Sc-PSMA-617 (4.89 ± 0.5 MBq) and imaged on a SPECT scanner at 4, 24, or 48 h postinjection. Mice were euthanized at 25 and 49 h time points, organs were collected, weighed and measured on gamma counter.

### Longitudinal Imaging and Therapy Study

LNCaP tumor-bearing mice were imaged with 0.8 nmol of [^43^Sc]­Sc-PSMA-617 (2.8 ± 0.2 MBq) at 1 h postinjection, at day −3 (3 days prior to administration of the therapeutic). After imaging, the mice were randomly assigned to one of three dosing groups in a blinded study. Mice were administered a dose of either 25.9 MBq [^47^Sc]­Sc-PSMA-617 (High dose: 10.8 nmol), 11.1 MBq [^47^Sc]­Sc-PSMA-617 (Low dose: 4.6 nmol), or saline (Control). Tumor measurements (mm) and animal weight (g) were recorded every other day. Mice were euthanized at the predefined end points: loss of 20% weight, tumor volume of >2500 mm^3^, or tumor ulcerations. After treatment administration, PET analysis of the predose scan was conducted. Post-treatment, the [^43^Sc]­Sc-PSMA-617 PET SUV_mean_ of each mouse was plotted against the end point to compare the PET SUV_mean_ to overall survival.

## Results

### Radiolabeling and Stability

Radioscandium PSMA-617 was prepared with >99% complexation. Representative iTLC and HPLC traces are shown in Figure S1 and S2. [^47^Sc]­Sc-PSMA-617 remains >99% intact throughout 14 d in human and mouse serum (Figure S2B). The molar activities of [^43^Sc]­Sc-PSMA-617 and [^47^Sc]­Sc-PSMA-617 were 7.7 ± 1.2 MBq/nmol and 2.4 ± 0.4 MBq/nmol, respectively. All complexes used in subsequent studies were >99% radiochemical purity.

#### *In Vitro* Studies

##### Saturation

[^47^Sc]­Sc-PSMA-617 saturation binding showed a *K*_d_ = 1.98 ± 0.01 nM, Figure 2A and Table S1.

**Figure 2:**
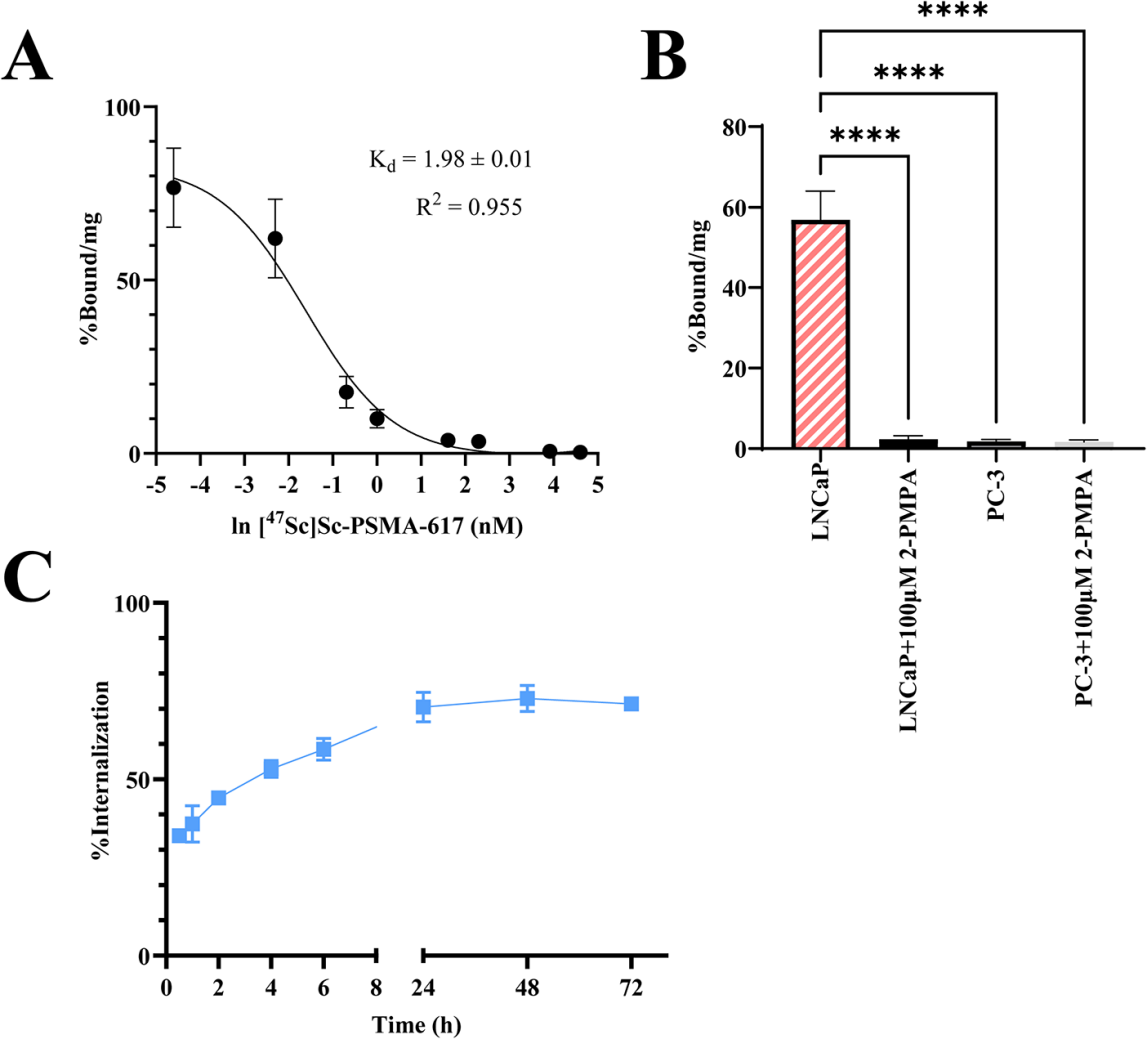
(A) The saturation binding curve of [^47^Sc]­Sc-PSMA-617 illustrates a *K*_d_ of 2 nM. (B) The cellular uptake of 1 nM [^47^Sc]­Sc-PSMA-617 in LNCaP, LNCaP+100 μM 2-PMPA, PC-3, and PC-3 + 100 μM 2-PMPA, analyzed using one-way ANOVA test. (C) The internalization of 1 nM [^47^Sc]­Sc-PSMA-617 in LNCaP cells up to 72 h. *p < 0.05, **p < 0.01, ***p < 0.001, ****p < 0.0001.

##### Cellular Uptake and Competitive Binding

All blocking studies used a concentration of 100 μM 2-PMPA, a known PSMA inhibitor.^46^ Cellular uptake studies using [^47^Sc]­Sc-PSMA-617 (1 nM) demonstrated significantly higher uptake (*P* < 0.0001) in LNCaP cells (56.8 ± 6.5% bound/mg) than blocked LNCaP cells (2.3 ± 0.7% bound/mg), PC-3 cells (1.8 ± 0.4% bound/mg), and in blocked PC-3 cells 1.6 ± 0.4% bound/mg) (Figure 2B and Table S2).

##### Internalization

The internalization of [^47^Sc]­Sc-PSMA-617 (1 nM) reached 70.5 ± 3.7% by 24h and remained stable to 72h (71.4 ± 1.1%). (Figure 2C and Table S3).

### *In Vivo* PET Studies with [^43^Sc]­Sc-PSMA-617

Mice implanted with LNCaP tumors were injected with 2.2 ± 0.6 MBq [^43^Sc]­Sc-PSMA-617 and imaged immediately with a dynamic 60 min scan. Images were reconstructed into six consecutive 10 min frames to determine circulation and uptake in the PSMA+ tumor. The average SUV_mean_ values over time of the heart, kidney, liver, bladder, tumor, and the tumor-to-heart ratio are shown in Figure S3A–F, with values given in Table S4.

#### *In Vivo* Specificity

Mice implanted with LNCaP or PC-3 tumors were injected with 2.2 ± 0.6 MBq [^43^Sc]­Sc-PSMA-617 or [^43^Sc]­Sc-PSMA-617 coinjected with 2-PMPA at 5 kg/mg, imaged at 1 h and biodistributions were conducted at 1.5 h with results shown in Figure 3. Representative maximum intensity projections (MIP) are shown in Figure 3A. The average tumor PET SUV_mean_ values are shown in Figure 3B, where the LNCaP SUV_mean_ (0.32 ± 0.03) was significantly higher (*P* < 0.0001) than both the blocked LNCaP group (0.02 ± 0.01) and PC-3 group (0.07 ± 0.01). Figure 3C shows the tumor biodistribution results, wherein the LNCaP group (2.2 ± 0.3) exhibited significantly higher uptake (*P* < 0.0001) compared to the blocked LNCaP group (0.5 ± 0.2) and the PC-3 group (0.3 ± 0.2). The biodistribution of select organs of the three groups is given in Figure 3D, and the complete biodistribution is shown in TABLE S5. The uptake of the kidneys in the LNCaP group (5.05 ± 1.4%ID/g) was significantly higher (*P* = 0.0246) than that of the blocked LNCaP group (0.62 ± 0.7%ID/g).

**Figure 3:**
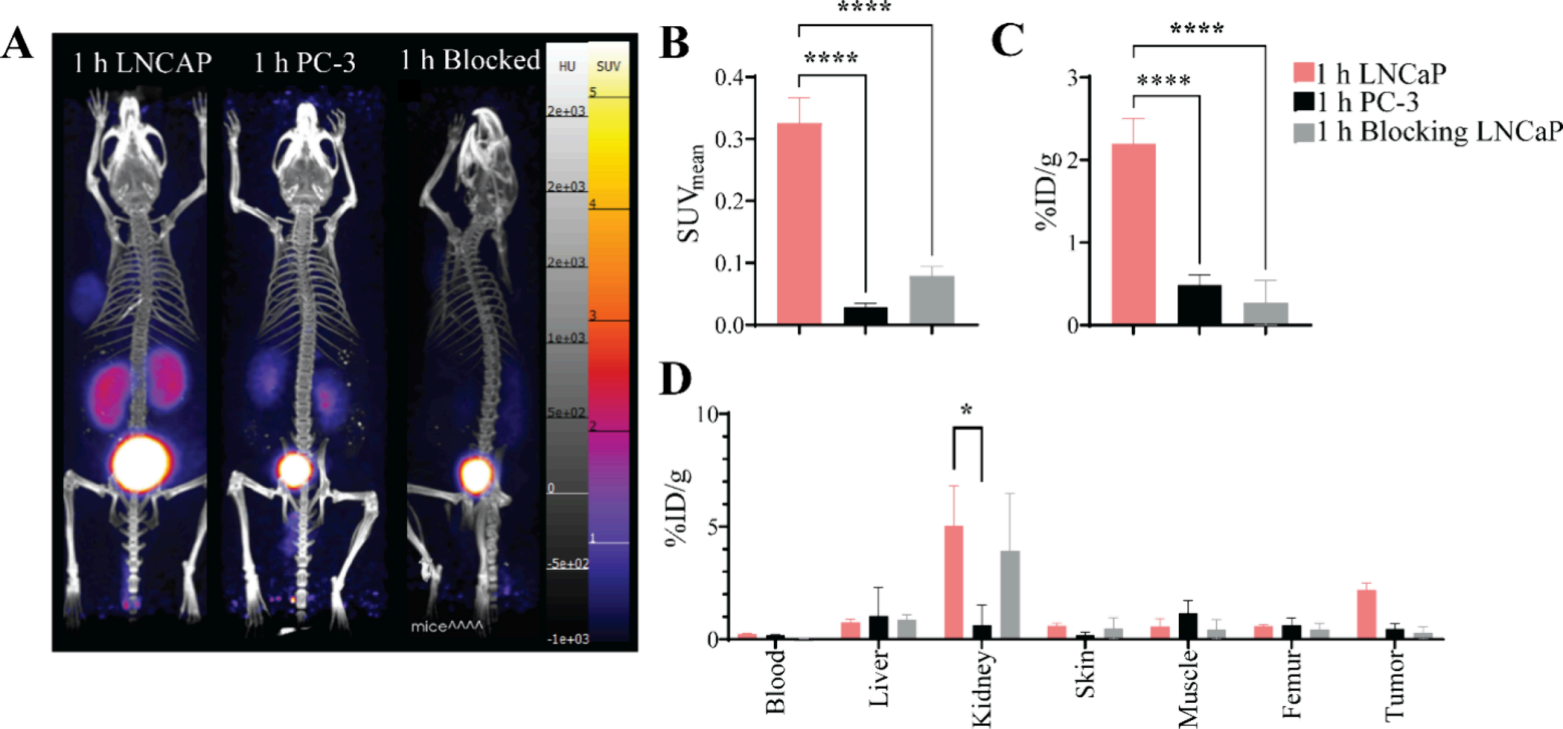
(A) MIPs [^43^Sc]­Sc-PSMA-617 PET scans of three groups at 1 h postinjection for LNCaP, PC-3 and blocked LNCaP tumor bearing mice. All images are windowed the same for comparison. (B) Tumor [^43^Sc]­Sc-PSMA-617 PET SUV_mean_ at 1 h postinjection for LNCaP, PC-3, and blocked LNCaP analyzed using a one-way ANOVA. (C) Tumor %ID/g biodistribution at 1.5 h for LNCaP, PC-3 and blocked LNCaP tumors analyzed using a one-way ANOVA. (D) The biodistribution of the three groups at 1.5 h postinjection. All groups were *n* = 4. *p < 0.05, **p < 0.01, ***p < 0.001, ****p < 0.0001.

#### Extended *In Vivo* Imaging

LNCaP tumor-bearing mice were injected with 2.2 ± 0.6MBq of [^43^Sc]­Sc-PSMA-617 and imaged at 1, 2, and 4 h, followed by biodistributions (Figure S4). Representative MIPs are shown in Figure S4A. The PET SUV_mean_ values of the 1 h LNCaP group (0.32 ± 0.03) were not significantly different than the 2 h group (0.31 ± 0.05), while the 4 h LNCaP group (0.48 ± 0.03) was found to be significantly higher (*P* = 0.0021) (Figure S4B). There were no significant differences in the radiopharmaceutical uptake (%ID/g) of the LNCaP tumors (Figure S4C) at 1, 2, or 4 h. The biodistribution of select organs of the three groups is shown in Figure S4D, represented as %ID/g, and the complete biodistribution is given in Table S6.

An extended period time activity curve was generated by imaging the same set of mice throughout 2 half-lives of ^43^Sc. LNCaP tumor-bearing mice were administered 1.6 ± 0.1MBq of [^43^Sc]­Sc-PSMA-617 and imaged at 1, 2.5, 4.5, 7, and 9 h postinjection. Representative MIPs are shown in Figure S5A. The average mean time activity curves of the bladder, kidney, tumor, and tumor-to-heart ratio are shown in Figure S5B, Figure S5C, Figure S5D, and Figure S5E respectively. All values for the time activity curves are provided in Table S7.

### Comparative Biodistribution of [^43^Sc]­Sc-PSMA-617 and [^68^Ga]­Ga-PSMA-617

A comparison of [^43^Sc]­Sc-PSMA-617 with [^68^Ga]­Ga-PSMA-617 was conducted in LNCaP tumor-bearing mice, injected with the same mass of PSMA-617 (0.3 nmol), imaged at 1 h postinjection, followed by biodistributions. Representative MIPs are shown in Figure 4A. There was no significant difference between the tumor SUV_mean_ of [^43^Sc]­Sc-PSMA-617 and [^68^Ga]­Ga-PSMA-617 (Figure 4B). The %ID/g ratios of the tumor-to-muscle, tumor-to-blood, tumor-to-kidney and tumor-to-liver are shown in Figure 4C where [^43^Sc]­Sc-PSMA-617 had higher tumor-to-muscle and tumor-to-liver ratios than [^68^Ga]­Ga-PSMA-617 (tumor-to-muscle: 4.9 ± 2.2 vs 1.4 ± 0.8; tumor-to-liver: 2.9 ± 0.3 vs 0.5 ± 0.2) while [^68^Ga]­Ga-PSMA-617 had a higher tumor-to-kidney ratio than [^43^Sc]­Sc-PSMA-617, 0.9 ± 0.3 vs 0.5 ± 0.1. The biodistribution of select organs of the two groups is shown in Figure 4D, and the complete biodistribution is given in Table S8. The %ID/g of the livers were significantly different (*P* = 0.00041) with [^68^Ga]­Ga-PSMA-617 (5.7 ± 0.6%ID/g) being substantially higher than [^43^Sc]­Sc-PSMA-617 (0.7 ± 0.1%ID/g). The %ID/g of the [^68^Ga]­Ga-PSMA-617 (2.5 ± 1.1%ID/g) in the tumor was not significantly different than the [^43^Sc]­Sc-PSMA-617 (2.2 ± 0.2%ID/g) tumor uptake.

**Figure 4:**
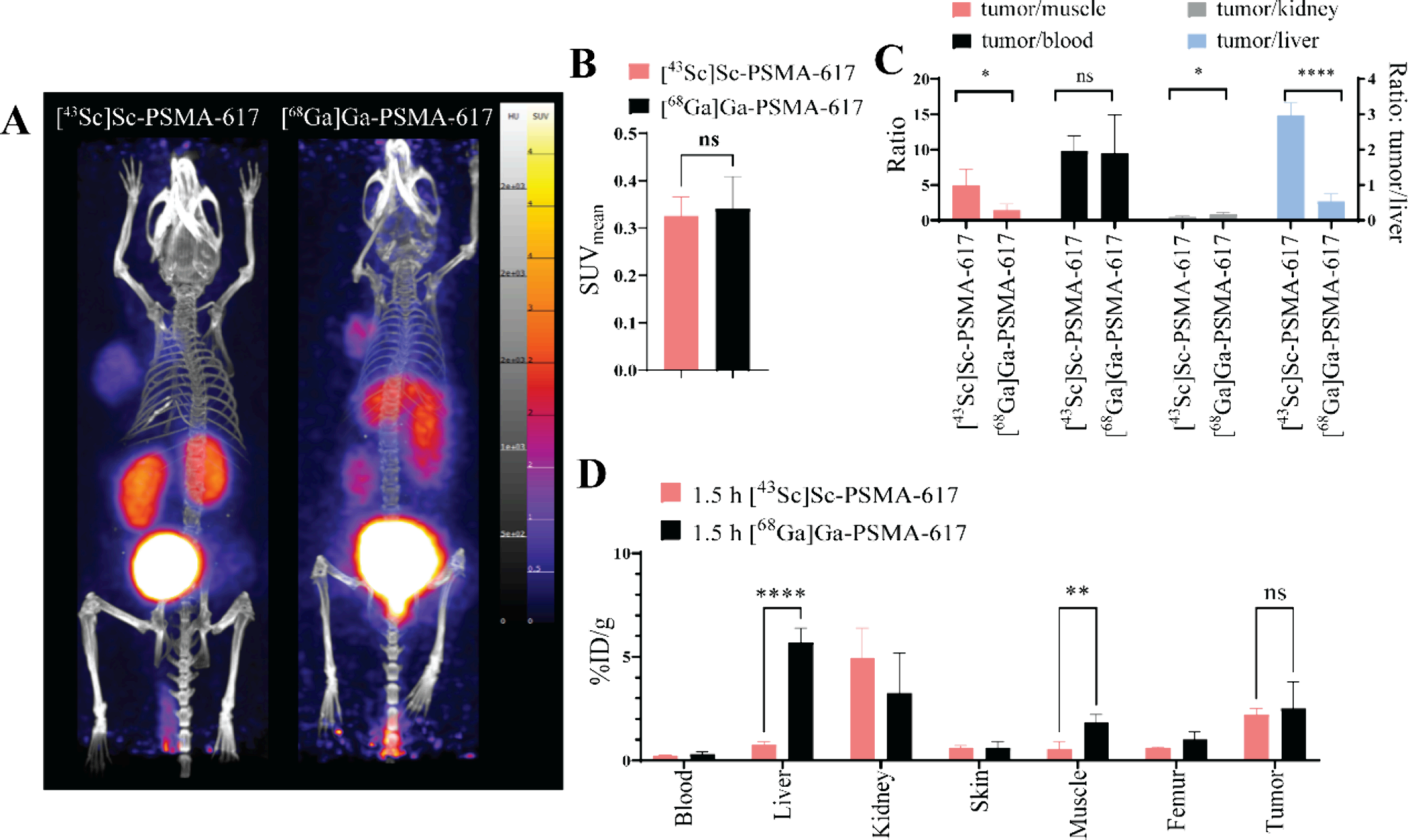
(A) MIPs of [^43^Sc]­Sc-PSMA-617 and [^68^Ga]­Ga-PSMA-617 PET scans in animals bearing LNCaP-tumors at 1 h postinjection. All images are windowed the same for comparison. B) Tumor PET SUV_mean_ comparison between the 1 h LNCaP of [^43^Sc]­Sc-PSMA-617 and [^68^Ga]­Ga-PSMA-17, analyzed using a *t* test. (C) The tumor-to-muscle, tumor-to-blood, tumor-to-kidney, and tumor-to-liver%ID/g ratios were analyzed using *t* tests. (D) The biodistribution of select organs at 1.5 h postinjection in animals bearing LNCaP tumors for [^43^Sc]­Sc-PSMA-617 and [^68^Ga]­Ga-PSMA-617, analyzed using *t* tests. All groups were *n* = 4. *p < 0.05, **p < 0.01, ***p < 0.001, ****p < 0.0001.

### *In Vivo* SPECT Imaging with [^47^Sc]­Sc-PSMA-617

For additional extended time point imaging using the longer half-life of ^47^Sc, mice bearing LNCaP tumors were injected with 4.89 ± 0.2 MBq of [^47^Sc]­Sc-PSMA-617, imaged at 4, 24, and 48 h with biodistribution conducted at 25 and 49 h (Figure 5A). There was no significant difference between SPECT SUV_mean_ values of the LNCaP tumors at the 4, 24, or 48 h time points (Figure 5B) or between the%ID/g ratios for tumor-to-muscle, tumor-to-blood, and tumor-to-kidney between 24 and 48 h (Figure 5C). The complete biodistribution of the mice bearing LNCaP tumors at 25 and 49 h is shown in Figure 5D, represented as %ID/g, and is given in Table S9. There was no significant difference between the 24 and 48 h uptake of the tumors, 2.4 ± 0.1%ID/g versus 2.1 ± 0.1%ID/g. Additionally, all organs except kidneys (0.1 ± 0.03%ID/g, 0.1 ± 0.02%ID/g) and large intestine (0.4 ± 0.26%ID/g, 0.14 ± 0.02%ID/g) had <0.01%ID/g. Importantly, all organs had a lower%ID/g than the tumors.

**Figure 5:**
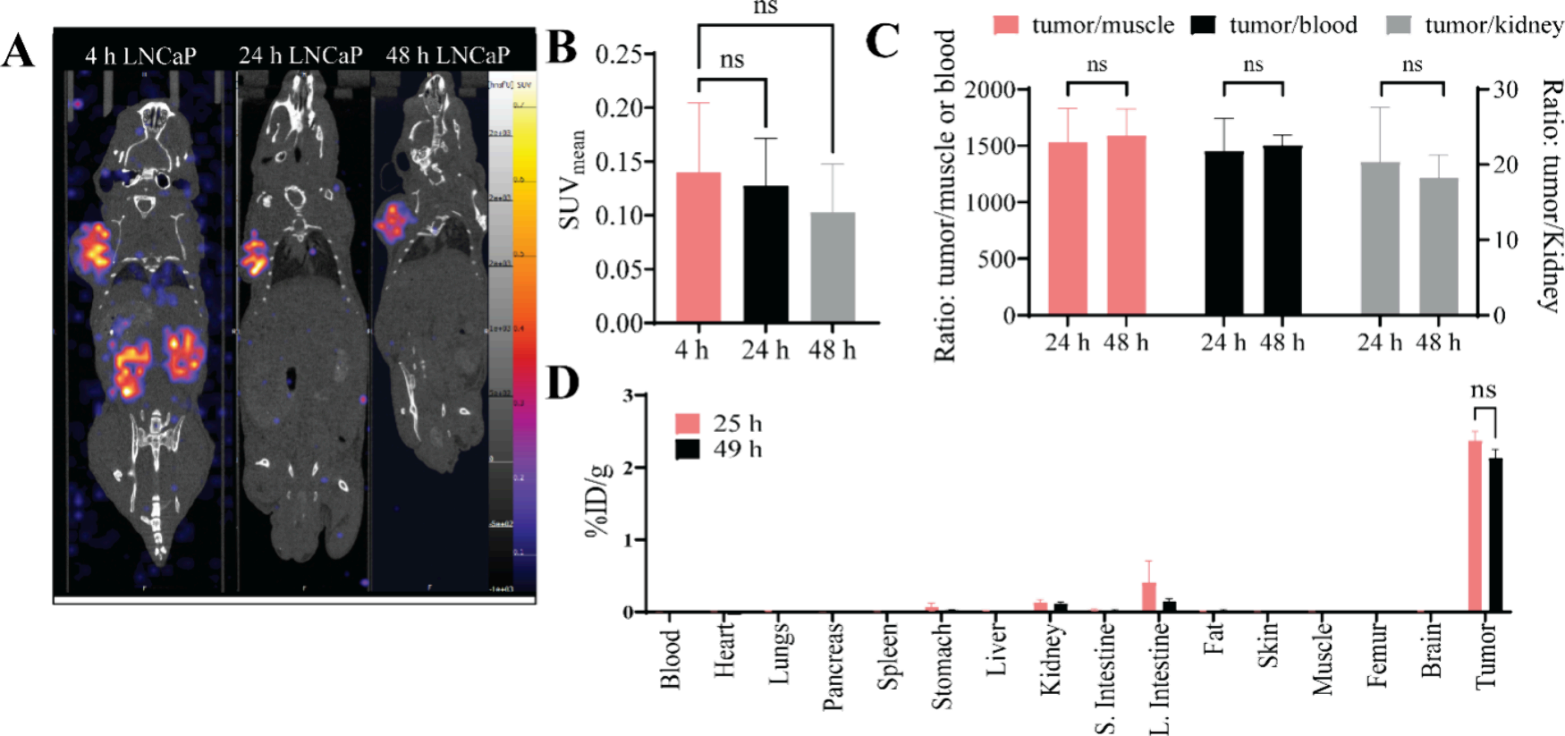
(A) A representative coronal section of [^47^Sc]­Sc-PSMA-617 SPECT scans of mice bearing LNCaP tumors at 4, 24, and 48 h postinjection. All images are windowed the same for comparison. (B) SPECT SUV_mean_ comparison for LNCaP tumors images at 4, 24, and 48 h, analyzed using a one-way ANOVA. (C) The%ID/g ratio comparison between tumor-to-muscle, tumor-to-blood, and tumor-to-kidney at 24 and 48 h, was analyzed using *t* tests. (D) The biodistribution of animals at 25 h (*n* = 4) and 49 h (*n* = 3) postinjection, represented as %ID/g, analyzed using *t* tests. *p < 0.05, **p < 0.01, ***p < 0.001, ****p < 0.0001.

### Longitudinal Imaging and Therapy Study

A longitudinal study was conducted in LNCaP tumor-bearing mice, where all animals were imaged with [^43^Sc]­Sc-PSMA-617 prior to being randomly sorted into three different therapy cohorts: high dose, low dose, or control (*n* = 5 per group). A Kaplan–Meier plot was used to evaluate the survival differences of the three groups, along with a Log-rank (Mantel-Cox) test for determining statistical differences between each treatment group. The average injection for the high dose group was 25.8 ± 0.9MBq of [^47^Sc]­Sc-PSMA (10.7 ± 0.4 nmol), for the low dose group was 11.1 ± 0.7MBq of [^47^Sc]­Sc-PSMA-617 (4.6 ± 0.3 nmol), or 100 μL of saline for the control. The median survival times were 62, 38, and 18 days for high dose, low dose, and control, respectively, as shown in the Kaplan–Meier plot in Figure 6A. Table 3 provides the survival results, where the P values for the Mantel-Cox were *P* < 0.0001. Average tumor volumes for each group are shown in Figure 6B and Table S10, where the control group showed a rapid increase in tumor volume, starting at d 8, while both [^47^Sc]­Sc-PSMA-617 dose levels illustrated delayed tumor growth, with an increase in tumor volume occurring at d 28 and 50, respectively. The average relative body weights for each group were maintained throughout the study, as the weight decrease did not exceed 20% as shown in Figure 6C and Table S11. Two mice developed ulceration, one from the control group and one from the low dose group. These mice were euthanized and treated as an end point.

**Figure 6:**
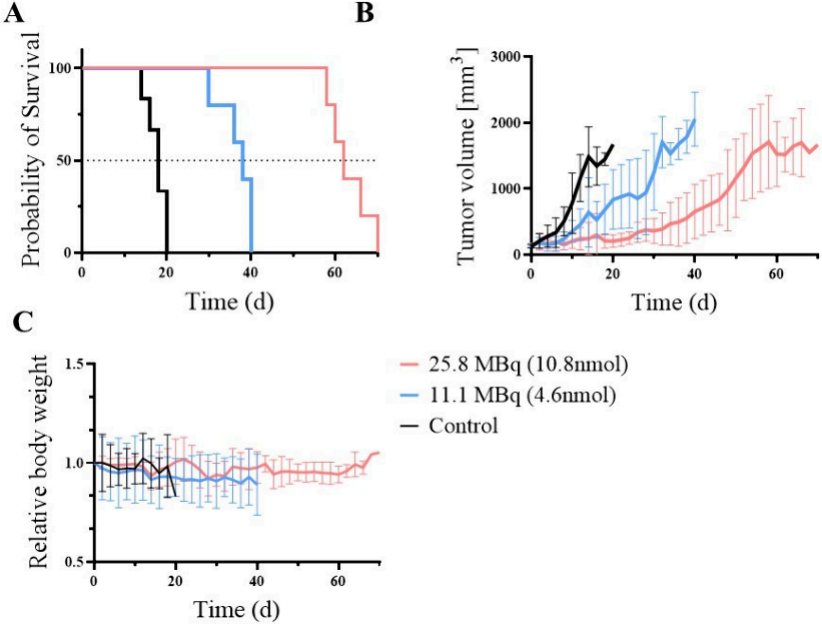
(A) Survival probability of the three therapy cohorts (high dose, low dose and control), (B) Tumor volume of the three therapy cohorts, (C) The relative body weight change of the three therapy cohorts.

**Table 3:** Results from the Theranostic Longitudinal Study

Cohort	Dose (MBq)	SUV_mean_	Median Survival (d)	Mantel-Cox Test *P* value
High dose (*n* = 5)	25.8 ± 0.9	1.55 ± 0.4	62	<0.0001
Low dose (*n* = 5)	11.1 ± 0.7	1.18 ± 0.1	38	
Control (*n* = 6)	0	1.28 ± 0.4	18	

[^43^Sc]­Sc-PSMA-617 tumor SUV_mean_ plotted against the survival time of the individual mice (d) from the high dose group (Figure 7A) and [^43^Sc]­Sc-PSMA-617 tumor SUV_mean_ plotted against the survival of the individual mice (d) from the low dose group (Figure 7B) show that these trends are linear with strong correlations, with *R* ^2^ values of 0.93 and 0.88 and Pearson r values of 0.96 and 0.94 for the high dose and low dose respectively (Table 3). Higher [^43^Sc]­Sc-PSMA-617 SUV_mean_ values were correlated to longer overall survival in both dose groups. A summary of the results from this theranostic study is shown in Table 3.

**Figure 7:**
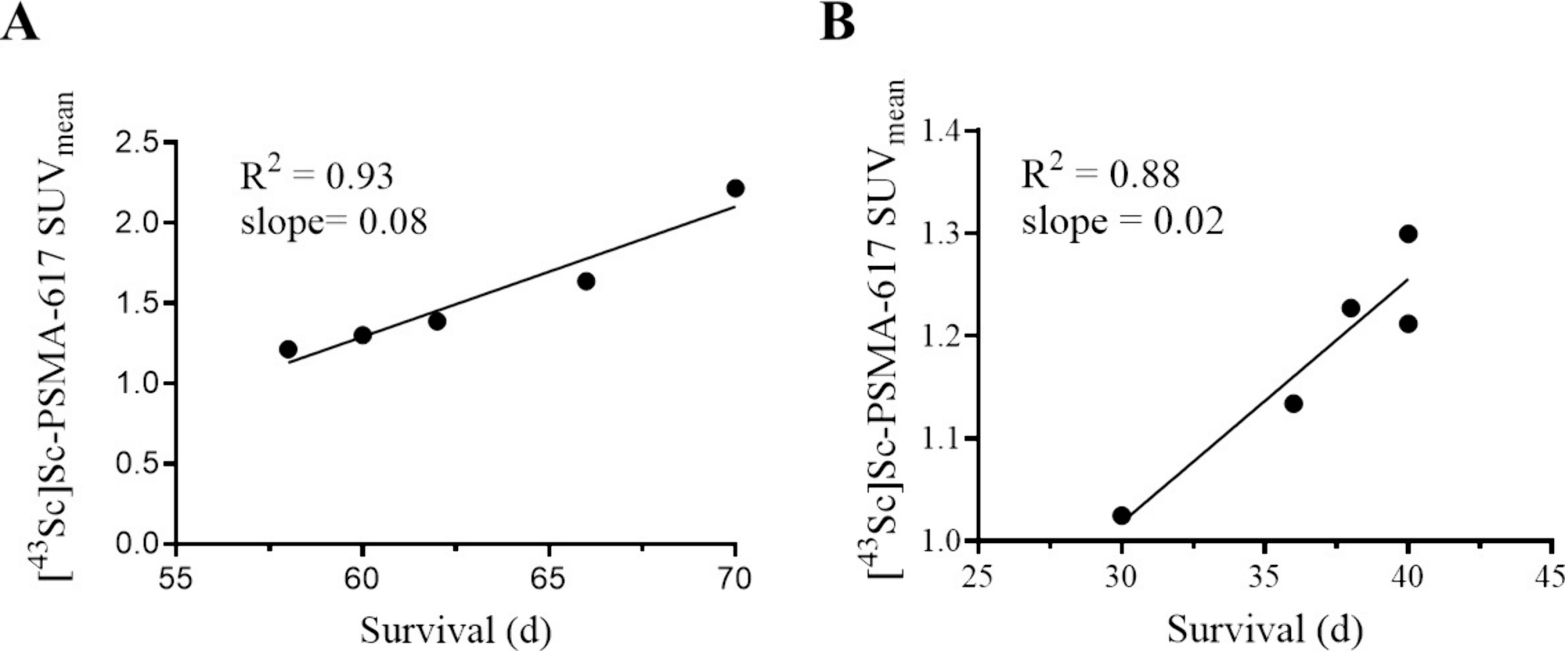
(A) Plot and linear regression of the SUV_mean_ values of 25.8 MBq (10.8 nmol) cohort vs the survival time of the 25.8 MBq (10.8 nmol) cohort. (B) Plot and linear regression of the SUV_mean_ of 11.1 MBq (4.6 nmol) cohort vs the survival time of the low dose cohort.

## Discussion

The clinical success of [^68^Ga]­Ga-PSMA-11 and [^177^Lu]­Lu-PSMA-617 has continued to push the expansion of the theranostic landscape, including the characterization of other radionuclides as potential theranostic pairs.^1,3,8^ Elementally matched pairs have significant advantages because the therapeutic compound has the same structure as the diagnostic compound, allowing scientists to directly determine the dosimetry and dosing strategies of the therapeutic agent, and potentially predict the therapeutic response based on the information from the diagnostic agent. The radioscandium nuclides have been proposed as an elementally matched theranostic pair, with much of the research focusing on their production and chelation. While these avenues continue to be explored, there is still a need to characterize ^43^Sc or ^44^Sc in conjunction with ^47^Sc, as a theranostic pair. This work demonstrates the theranostic potential of the radioscandium nuclides by incorporating them into the validated PSMA-617 compound for a theranostic study.

Both [^43^Sc]­Sc-PSMA-617 and [^47^Sc]­Sc-PSMA-617 were synthesized in high radiochemical purity (>99% purity), showing long-term stability (>99%) throughout 14 d, which is more than four times longer than the ^47^Sc half-life. The *K*_d_ of [^47^Sc]­Sc-PSMA-617 (1.98 ± 0.01 nM) closely aligns with literature values for [^44^Sc]­Sc-PSMA-617, [^177^Lu]­Lu-PSMA-617, [^68^Ga]­Ga-PSMA-617 and [^89^Zr]­Zr-PSMA-617, ranging from 2 to 12 nM.^10,47,48^ The *K*_d_ results, coupled with the cellular binding studies, demonstrate that [^47^Sc]­Sc-PSMA-617 has excellent cell binding and exhibits high internalization within 24 h in LNCaP cells. [^47^Sc]­Sc-PSMA-617 also showed specificity to the PSMA receptors, having a higher uptake in LNCaP cells compared to PC-3 and blocked-LNCaP cells. The internalization of [^47^Sc]­Sc-PSMA-617 reached a maximum within 24 h and was shown to be retained in the cell throughout 72 h, with retention in the tumor cell a requirement for effective radiotherapy.

LNCaP tumor-bearing mice imaged with [^43^Sc]­Sc-PSMA-617 showed tumor uptake, decreasing SUV_mean_ in the heart, kidneys, and liver over 60 min, and increasing uptake in the bladder, indicating excretion of the complex through the renal system. Importantly, the liver SUV_mean_ decreased over time; an organ is shown in the literature to have unassociated or “free” Sc uptake.^12,17,25^ These results strongly align with the published [^44^Sc]­Sc-PSMA-617 studies, showing specificity to PSMA+ tumors *in vivo* and clearance through renal excretion.^10,39^

Three different cohorts were assessed with [^43^Sc]­Sc-PSMA-617, LNCaP tumor-bearing mice, PC-3 tumor-bearing mice, and LNCaP tumor-bearing mice coadministered a dose of the PSMA inhibitor 2-PMPA (blocked). [^43^Sc]­Sc-PSMA-617 was shown to have significantly (*P* < 0.0001) higher uptake in the LNCaP tumors (2.2 ± 0.3%ID/g) than in the blocked LNCaP (0.5 ± 0.2% ID/g) and PC-3 tumors (0.3 ± 0.2%ID/g), demonstrating binding specificity to PSMA.

Due to the half-life of [^43^Sc]­Sc, which is 3.89 h, longer PET scan time points were conducted in two ways. Three groups were imaged at 1, 2, or 4 h, with biodistribution studies following to confirm the PET data. A second time-activity curve was generated from one cohort imaged multiple times over two half-lives. All imaging time points demonstrated that [^43^Sc]­Sc-PSMA-617 shows uptake in the LNCaP tumor, which is retained for over 8 h (ranging from 0.14 to 0.12 SUV) and is cleared from other nontarget tissues. This yields two significant implications: Sc-PSMA-617 is retained in the tumor, which is essential for the therapeutic effectiveness of [^47^Sc]­Sc-PSMA-617, and [^43^Sc]­Sc-PSMA-617 can be used for imaging at longer time points than [^68^Ga]­Ga-PSMA-11. These extended imaging time points may be advantageous for detecting metastases near clearance organs, such as the kidney and bladder. This also aligns closely with the study by Eppard et al., which utilized [^44^Sc]­Sc-PSMA-617.^39^

[^43^Sc]­Sc-PSMA-617 (2.2 ± 0.3%ID/g) did not significantly differ from [^68^Ga]­Ga-PSMA-617 (2.5 ± 1.1%ID/g) LNCaP tumor uptake, aligning with previous literature.^10^ The lower uptake of [^68^Ga]­Ga-PSMA-617 in this study compared to the literature may be due to the time-point difference, as Umbricht et al. reported biodistribution data at 2 h rather than 1 h.^10^ The significantly higher uptake of [^68^Ga]­Ga-PSMA-617 in the liver compared to [^43^Sc]­Sc-PSMA-617 also aligns with the literature, where reports have shown that [^68^Ga]­Ga-PSMA-617 exhibits higher liver uptake compared to [^44^Sc]­Sc-PSMA-617.^10^ Lastly, in Umbricht et al, both [^68^Ga]­Ga-PSMA-617 and [^68^Ga]­Ga-PSMA-11 were shown to have tissue distribution that significantly varied from [^177^Lu]­Lu-PSMA-617 while [^44^Sc]­Sc-PSMA-617 was nearly identical to [^177^Lu]­Lu-PSMA-617.^10^ Two conclusions can be drawn from this study and from Umbricht et al.: that [^43^Sc]Sc or [^44^Sc]Sc can be use in place of [^68^Ga]Ga as a more chemically similar and later imaging diagnostic agent with [^177^Lu]Lu and that using an elementally matched theranostic pair would eliminate any tissue differences between the diagnostic and therapeutic agent due to the production of identical complexes, given that they are produced with similar molar activities. This study demonstrated the importance of elementally matched theranostic compounds, as changing the radiometal in the PSMA-617 complex resulted in a change in the biodistribution of the complex.

The [^47^Sc]­Sc-PSMA-617 compound was evaluated at longer time points using SPECT imaging and biodistribution studies. There was no significant difference in the PSMA+ tumor uptake of [^47^Sc]­Sc-PSMA-617 from 24-to-48 h, indicating that the therapeutic compound is retained, which maximizes the dose delivered to the tumor. Additionally, the [^47^Sc]­Sc-PSMA-617%ID/g in all other organs was significantly lower than in the tumor, with the large intestine exhibiting the highest uptake among nontumor organs. The results are similar to those reported by Kuo et al., who investigated [^177^Lu]­Lu-PSMA-617 in mice bearing LNCaP tumors. [^47^Sc]­Sc-PSMA-617 and [^177^Lu]­Lu-PSMA-617 exhibit similar biodistribution, with a high clearance from the blood by 24 h, with <0.01 ± < 0.0.1%ID/g vs 0.00 ± 0.00, the kidneys at 0.13 ± 0.04%ID/g vs 0.58 ± 0.22%ID/g, and the heart at <0.01 ± < 0.0.1%ID/g vs 0.01 ± 0.00%ID/g. There is also similar uptake at 24 h in the liver, 0.02 ± < 0.01%ID/g vs 0.03 ± 0.01%ID/g, pancreas at <0.01 ± < 0.0.1%ID/g vs 0.01 ± 0.00%ID/g, and spleen at 0.1 ± < 0.1%ID/g vs 0.05 ± 0.01%ID/g. Both the tumor/muscle and tumor/kidney ratios are similar at 1532 ± 300 vs 1582 ± 353 and 20.4 ± 7.2 vs 20.7 ± 8.4, respectively. Additionally, by 24 h, both compounds have largely cleared from the system but show retention in the tumor. The largest difference is the absolute uptake in the tumor, at 2.37 ± 0.1%ID/g vs 10.9 ± 3.3% ID/g for the Sc and Lu compounds, respectively. This is largely attributed to the specific activity of the produced ^47^Sc and ^177^Lu. In this study. [^47^Sc]­Sc-PSMA-617 was synthesized at 2.4 ± 0.4 MBq/nmol whereas Kuo et al. reports 782 ± 43.3 GBq/μmol. As ^47^Sc production methods continue to improve and yield higher batch activities, the molar activities will likely increase, as well as ^47^Sc accessibility.

A blinded longitudinal theranostic study was conducted to determine if the [^43^Sc]­Sc-PSMA-617 PET results can be correlated with the therapeutic response to [^47^Sc]­Sc-PSMA-617 in LNCaP tumor-bearing mice. Two dose levels of [^47^Sc]­Sc-PSMA-617 were investigated, 25.9 MBq and 11.1 MBq, and an additional group was administered saline as a control. There was a significant response for both [^47^Sc]­Sc-PSMA-617 dose levels, as evidenced by prolonged survival and delayed tumor growth. The high dose group responded better than the low dose group, with longer median survival. Additionally, the Mantel-Cox test showed a significant difference between the survival plots of all three cohorts, indicating again there is a delayed tumor growth observed with increased administered [^47^Sc]­Sc-PSMA-617 activity. The results are similar to those reported by Kuo et al. using various ^177^Lu labeled PSMA compounds, including PSMA-617 at 18.9 ± 0.9 MBq, which resulted in a median survival d of 58 with the longest survival at 74 d.^47^ The mean β- energy of ^177^Lu, (E_β‑mean_ = 133 keV) is comparable to the mean β- energy of ^47^Sc, (E_β‑mean_ = 162 keV), and can elicit a comparable response as shown in Siwowska et al.^41^ The increased dose at 25.9 MBq given here compared to the 18.9 MBq in Kou et al. was used so that this study will have the same number of ^47^Sc decays at the tumor site as the ^177^Lu study reported in Kou et al.^47^

The relationship between SUV_mean_ of [^43^Sc]­Sc-PSMA-617 and survival time for both [^47^Sc]­Sc-PSMA-617 dose groups showed a strong linear correlation, indicating that a higher SUV_mean_ resulted in longer survival. Since [^47^Sc]­Sc-PSMA-617 is chemically identical to [^43^Sc]­Sc-PSMA-617, a higher uptake of the diagnostic compound would imply a higher uptake of the therapeutic compound, leading to improved responses. It is also interesting to note that the relationship is different depending on the dose level as the two cohorts have different slopes (0.08 SUV_mean_/d for high dose group and 0.02 SUV_mean_/d for low dose group). The SUV_mean_ vs the survival date is not linear when data is combined from both dose groups, with an *R* ^2^ of 0.56, another indication that this prediction is specific for each of the dose levels in our study. This should be carefully considered for all theranostic studies, especially when comparing results from different sites using different dosing strategies. Future studies should continue to explore this by exploring additional doses at lower and higher activity in conjunction with PET imaging.

The finding that [^43^Sc]­Sc-PSMA-617 predicts the therapeutic response of [^47^Sc]­Sc-PSMA-617 supports the use of radioscandium nuclides as a theranostic pair. Future studies can explore multiple doses of [^47^Sc]­Sc-PSMA-617 based on PET-derived dose schemes and timing as well as toxicity studies post therapy. Additionally, dosimetry studies for both [^43^Sc]­Sc-PSMA-617 and [^47^Sc]­Sc-PSMA-617 would inform on optimization of dosing schemes and may provide further insights into the potential advantage of using an elementally matched theranostic pair. Lastly, chemically identical diagnostic radiopharmaceuticals could be used to determine the dosimetry of therapeutic complexes, predict therapeutic responses, and enable precision personalized medicine through the continued use of diagnostic PET scans to monitor and improve response to targeted radioscandium therapy.

## Conclusions

Overall, this study yielded two chemically identical PSMA-targeting scandium radiopharmaceuticals with high purity, demonstrating both stability and *in vivo* specificity to PSMA. A theranostic longitudinal study, where PSMA+ tumor bearing mice were imaged with [^43^Sc]­Sc-PSMA-617 before receiving a [^47^Sc]­Sc-PSMA-617 dose, illustrated that two different [^47^Sc]­Sc-PSMA-617 dose levels delayed tumor growth compared to the control. The PET SUV_mean_ from [^43^Sc]­Sc-PSMA-617 images was shown to predict the response of [^47^Sc]­Sc-PSMA-617 with a higher PET SUV_mean_ correlating with longer survival in animals with the same genetic background implanted with the same tumor cell line. This study demonstrated a proof-of-concept theranostic study showing that ^43^Sc and ^47^Sc can be used as an elementally matched theranostic pair.

## Supplementary Material



## Notes

Detailed descriptions of the experimental procedures, including materials, radioscandium production, radiolabeling protocols, *in vitro* experiments, *in vivo* PET, SPECT and therapeutic studies, imaging parameters and analysis methods, and statistical analysis. Results of the radiolabeling studies, including iTLC and HPLC chromatograms of the radiocomplexes, serum stability data, and the tabulated results for the *in vitro* saturation binding, cellular uptake, internalization and competitive binding assays. Results from the time activity curves of the 60 min dynamic PET scan, the results from the time activity curves at 1, 2.5, 4.5, 7, and 9 h, the tabulated biodistribution data of [^43^Sc]­Sc-PSMA-617 in mice bearing LNCaP tumors, PC-3 tumors and LNCaP tumors coinjected with 2-PMPA, as well as the tabular biodistribution results for ^43^Sc]­Sc-PSMA-617 and [^68^Ga]­Ga-PSMA-617. The tabular results of the *in vivo* SPECT imaging with [^47^Sc]­Sc-PSMA-617, the equation to determine the tumor volume (mm^3^), tabulated average tumor volume (mm^3^) per cohort, and average relative weight change of the three cohorts (PDF)
