# Supplementary material for: Investigating the Theranostic Potential of Elementally Matched [43Sc]Sc-PSMA-617 and [47Sc]Sc-PSMA-617
Source: Mol Pharm. 2026 Feb 18;23(3):1517–28. doi: 10.1021/acs.molpharmaceut.5c01023 (PMC12958282; doi:10.1021/acs.molpharmaceut.5c01023)
Supplement: Supplementary file 1 [file mp5c01023_si_001.pdf]

Supplemental Information: Investigating the theranostic potential of elementally matched $[^{43}Sc]$Sc-PSMA-617 and $[^{47}Sc]$Sc-PSMA-617

Shelbie J. Cingoranelli[a,b#], Emily Putnam[a,b], Hailey Houson[a], Grayson Gimblet[a], Sharon Samuel[a], Volkan Tekin[a], Suzanne E. Lapi[a,b*]

[a]Department of Radiology, University of Alabama at Birmingham, Birmingham, AL, 35233

[b]Department of Chemistry, University of Alabama at Birmingham, Birmingham, AL, 35233

* Corresponding author; E-mail: lapi@uab.edu. Telephone (205)975-8689. Address 1924 6th Ave. S., WTI 310F, Birmingham, AL 35244.

[#]First author; E-mail: sjcing@uab.edu. Telephone (205)996-0894. Address 1824 6th Ave. S,. WTI301A, Birmingham, AL 35233. (Graduate Student)

The UAB cyclotron facility is a member of the Department of Energy University Isotope Network and is supported through DESC0021269 (PI: Lapi). Small animal imaging studies were supported by the O'Neal Cancer Center Grant P30CA013148. The authors declare no known competing financial or personal interests.

Details of the experiments, additional information including iTLC traces of the radiocomplexes, the *in vitro* results, the biodistribution tables, and the time activity tables are given within the supplementary material.

Materials and Methods

All chemicals were trace metal grade or analytical. Water was obtained from an 18.2 MΩ-cm water purification system (Milli-Q System, Millipore, Billerica, MA) and interacted with Chelex resin (Bio-Rad, Hercules, CA) for 24 h prior to filtration. PSMA-617 was ordered from Medkoo Biosciences (Morrisville, NC) and dissolved in water at 5 mg/mL concentrations. The High-Performance Liquid Chromatography (HPLC), system utilized an Xterra reversed-phase C-18 column (5 µm, 150 × 4.6 mm), purchased from Waters (Milford, MA). Radio-HPLC was performed using an Agilent 1200 series HPLC (Lexington, MA) with an in-line NaI(Tl) detector, using H_2O with 0.1% trifluoracetic acid (TFA) and acetonitrile with 1% TFA. Radio thin-layer chromatography (radio-iTLC) was performed with Eckert & Ziegler AR-2000 radio-TLC scanner (Hopkinton, MA) and glass microfiber chromatography paper impregnated with silica-gel (iTLC-SG) (Agilent, Santa Clara, CA). All cell cultures, cell culture media (Gibco RPMI 1640 and DMEM), Gibco fetal bovine serum, and Gibco trypsin-EDTA (0.25% trypsin/0.53 mM EDTA) were purchased from ATCC (Manassas, VA). Ammonium acetate (99.999%) was purchased from Sigma-Aldrich (St. Louis, MO). Glacial acetic acid, EDTA, acetonitrile, NaCl, and HCl (99.999%) were purchased from Fisher Scientific (Waltham, MA). Gallium-68 was eluted from a $^{68}Ge/^{68}Ga$ generator (Eckert & Ziegler, Santa Clara, CA) using 0.1 M HCl and trapped on a SCX cartridge (Agilent Lexington, MA) and eluted with 200 µL 4.8 M NaCl/0.1 M HCl solution. PET/CT imaging studies were performed on Sofie GNEXT scanner (Sofie Bioscience, CA, USA). SPECT images were obtained from a U-SPECT^6-µCT-OI scanner

(MILabs, Utrecht, The Netherlands). An automated gamma counter (Hidex AMG, Turku, Finland) was used for all biodistributions for both weight and activity measurements.

Radioscandium production

Scandium-43 and ^{47}Sc were produced as previously reported in Cingoranelli et al. (*1-3*) Briefly, enriched [^{46}Ti]TiO_2 was irradiated with 18 MeV protons at 20 μA for 2 h for the production of [^{43}Sc]Sc. [^{43}Sc]Sc was purified from the titanium target using BDGA chromatography and obtained in 0.1M HCl. Natural vanadium targets were irradiated with 24 MeV protons at 65 μA for 8 h for the production of [^{47}Sc]Sc. [^{47}Sc]Sc was purified from the vanadium target using MP-50 resin chromatography, followed by CM chromatography to obtain [^{47}Sc]Sc in 2M HCl. All produced radioscandium was evaporated to dryness using a Smart Evaporator in a 5 mL PFA vial before being reconstituted in 20 μL of 0.05 M HCl.

Radiolabeling

Radioscandium was incorporated into the PSMA-617 compound by incubation of 5-20 μL of ^{43}Sc or ^{47}Sc in 0.05M HCl, 50-75 μL 0.25M ammonium acetate at pH 4, at 95°C, 800 RPM, for 30 min. Radiocomplexation was first analyzed by HPLC, which was used to validate an instant thin-layer chromatography (iTLC) method. The HPLC gradient was 95% water with 0.1% TFA/5% acetonitrile with 0.1% TFA to 20% water with 0.1% TFA / 80% acetonitrile with 0.1% TFA over 15 min, at 0.7 mL/min flow rate. The mobile phase of the iTLC strips was 50 mM EDTA, pH 5.5, where Sc-PSMA-617 has an RF of 0.3 while $ScCl_3$ has an RF of 1, with representative iTLC traces shown in **FIGURE S1.**

***In vitro* studies**

***In vitro* stability:** For stability studies, [^{47}Sc]Sc-PSMA-617 was incubated in either human or mouse serum up to 14 d.

Saturation binding: LNCaP cells were incubated with 1 mL of media containing [^{47}Sc]Sc-PSMA-617 from 0.01 – 100 nM concentrations for 2 h at 37°C. The cells were then washed with PBS and lysed with 0.2 M NaOH, and the lysate was measured using a gamma counter. A bicinchoninic acid assay (BCA) was performed to measure the total protein concentration.

Cellular uptake and competitive binding: For competitive binding assays, 1 mL of media containing 1 nM of [^{47}Sc]Sc-PSMA-617 was added to wells containing either LNCaP cells, LNCaP cells with 100 µM 2-PMPA, PC-3 cells, or PC-3 cells with 100 µM 2-PMPA and incubated for 1 h at 37°C. The cells were then washed with PBS and lysed with 0.2 M NaOH, and the lysate was measured using a gamma counter. A BCA was performed to measure the total protein concentration.

Internalization: LNCaP cells were incubated with 1 mL of media containing [^{47}Sc]Sc-PSMA-617 at a concentration of 1 nM for 0.5, 1, 2, 4, 6, 24, 48, and 72 h at 37 °C. A 400 µL of 0.1 M cold citric acid was added to each well and incubated at room temperature (RT) for 5 minutes before removal. After the addition of citric acid, the cells were then lysed with 0.2 M NaOH. Both the citric acid solution and lysate were measured separately on a gamma counter. The results were analyzed by calculating the percentage of activity in the lysed portion relative to the total activity, which is the sum of the citric and lysate fractions.

***In vivo* PET studies with [^{43}Sc]Sc-PSMA-617**

***In vivo* stability:** To determine [^{43}Sc]Sc-PSMA-617 circulation and uptake in the PSMA+ tumor, LNCaP-bearing mice were injected with 0.2-0.3 nmol of [^{43}Sc]Sc-PSMA-617 and immediately underwent a 60 min dynamic scan which was reconstructed in 10 min frames.

***In vivo* specificity:** LNCaP tumor-bearing mice were injected with 0.3 nmol of [^{43}Sc]Sc-PSMA-617, or were co-injected with 0.3 nmol of [^{43}Sc]Sc-PSMA-617 and 2-PMPA at 5 mg/kg, and PC-3 tumor-bearing mice were injected with 0.3 nmol of [^{43}Sc]Sc-PSMA-617. All animals were imaged at 1 h post injection. After imaging, mice were euthanized, organs were collected, weighed and measured on a gamma counter.

Extended *in vivo* imaging: LNCaP-bearing mice were injected with 0.2-0.8 nmol of [^{43}Sc]Sc-PSMA-617 and imaged at either 1, 2 or 4 h. After imaging at either 1, 2 or 4 h, mice were euthanized, organs were collected, weighed and measured on a gamma counter.

LNCaP-bearing mice were injected with 0.2-0.8 nmol of [^{43}Sc]Sc-PSMA-617 and imaged at 1, 2.5, 4.5, 7 and 9 h. After the 9 h imaging timepoint, mice were euthanized, and organs were collected, weighed, and measured on a gamma counter.

Longitudinal imaging and therapy study

LNCaP tumor-bearing mice were imaged with 0.8nmol of [^{43}Sc]Sc-PSMA-617(2.8±0.2 MBq) at 1 h post injection. After imaging, the mice were randomly assigned to one of three dosing groups in a blinded study, with PET analysis performed only after treatment administration. The PET imaging was conducted 3 d prior to administering a dose of either 25.9 MBq [^{47}Sc]Sc-PSMA-617 (High dose: 10.8 nmol), 11.1 MBq [^{47}Sc]Sc-PSMA-617 (Low dose: 4.6 nmol) or saline (Control). Tumor measurements (mm) and animal weight (g) were collected every other day. Mice were euthanized at the predefined endpoints: loss of 20% weight, tumor volumes at

>2500mm^3, or the tumor became necrotic (skin broken down or ulcerations). Weight changes were determined by comparing the daily weight to the starting weight measured on the day of [^{47}Sc]Sc-PSMA-617 administration. The [^{43}Sc]Sc-PSMA-617 PET SUV_{mean} of each mouse was plotted against survival time.

Imaging parameters and analysis:

PET imaging parameters: All static PET scans had a 3.5 min CT scan immediately after PET acquisition. A Sofie small animal PET scanner was used for all PET images. The PET energy window was 350–650 keV. All images were reconstructed via the 3D-OSEM (Ordered Subset Expectation Maximization) algorithm (24 subsets and 3 iterations) with random, attenuation, and decay correction. The CT voltage was 80 kVp, current 150 μA, 720 projections, scan time 3.5 min, and was reconstructed with the Modified Feldkamp Algorithm.

SPECT imaging parameters: All static SPECT scans had a 5 min CT scan immediately after SPECT acquisition. A U-SPECT^6-μCT-OI scanner with a General Purpose Rat and Mouse collimator (MILabs, Utrecht, The Netherlands) was used for all SPECT acquisitions, and MIlabs software was used to reconstruct, register, and attenuation correct all SPECT data. All SPECT scans were acquired in listmode and reconstructed as a 1 h long, static scan at 0.8 voxels with 1.0 Gaussian filter, using 128 subsets and 20 iterations. Energy window 140-170 keV. The CT voltage was 20 kV, 0.07 mA, 720 projections, scan time 5 min, and reconstructed with the MILabs software. PMOD (PMOD Technologies Ltd., Adliswil, Switzerland) software was used to convert the software to DICOM format.

Post reconstruction image analysis: After the images were reconstructed, the PET or SPECT images were appended to the CT images and analyzed using *VivoQuant 4.0* (Invicro Imaging

Service and Software, Boston MA) software. Regions of interest were drawn for select tissues in each mouse for all images based on the CT images. The time-activity curves of the tracer were generated over the course of data collection for mice that underwent multiple scans.

Statistics

All data were analyzed using GraphPad Prism 9.4, with independent t-tests, one-way ANOVA tests, the Mantel-Cox test, the Gehan-Breslow-Wilcoxon test, simple linear regressions, or the Pearson Correlation Coefficient, as detailed in figure captions.

Results

In vitro analysis:

Radiolabeling

The radionuclidic purity of [^{43}Sc]Sc or [^{47}Sc]Sc was ≥98%. All complexes used in the following studies had a complexation of >99%. Representative iTLC scans are shown in **SUPPLEMENTAL FIGURES S1A** and **S1B**. Both [^{43}Sc]Sc-PSMA-617 and [^{47}Sc]Sc-PSMA-617 had an HPLC retention of 8.5 min while, free Sc retention was 2 min, shown in **FIGURE S2A**, The [^{47}Sc]Sc-PSMA-617 remained >99% intact throughout 14 d in both human and mouse serum, as shown in **FIGURE S2B**. The specific activity of [^{43}Sc]Sc-PSMA-617 and [^{47}Sc]Sc-PSMA-617 was 7.7 ± 1.2 MBq/nmol and 2.4 ± 0.4 MBq/nmol, respectively.

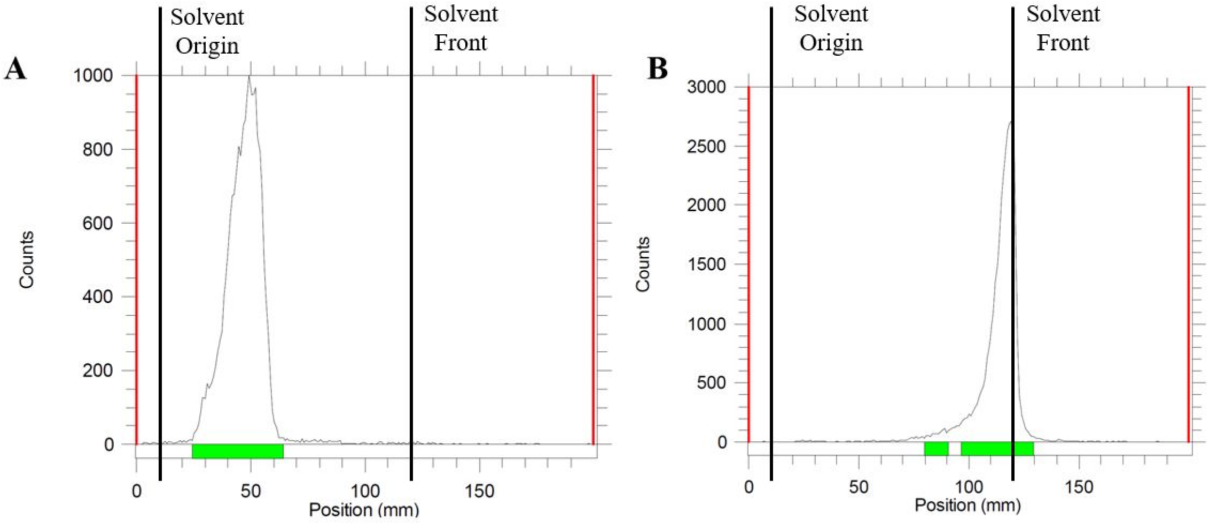

FIGURE S1. The iTLC traces for (A) [^{47}Sc]Sc-PSMA-617 and (B) [^{47}Sc]$ScCl_3$.

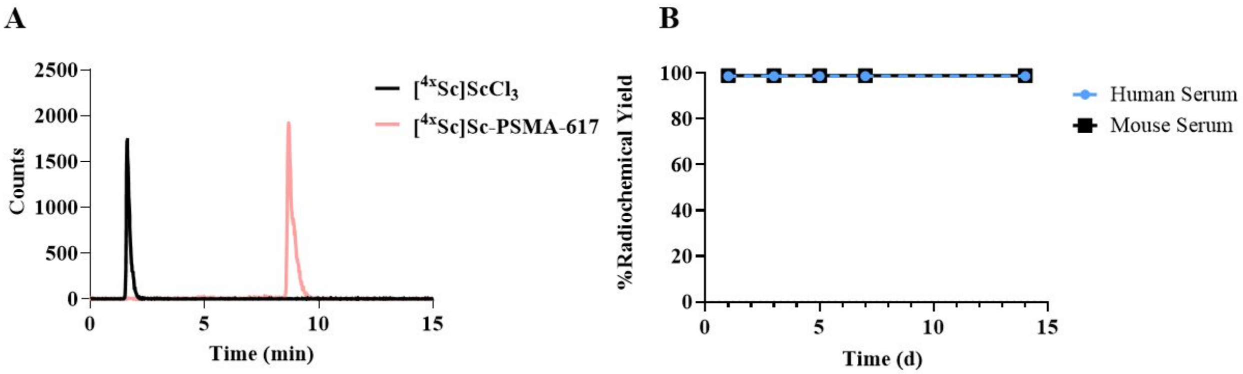

FIGURE S2 (A) Radio-HPLC traces showing [^{43}Sc]Sc-PSMA-617 (pink) and [^{43}Sc]$ScCl_3$ (black). (B) The stability of [^{47}Sc]Sc-PSMA-617 is represented as the mean ± SD of %. Radiochemical purity in human serum (blue circles) and mouse serum (black squares) throughout 14 d.

Saturation:

TABLE S1. The saturation binding results of [^{47}Sc]Sc-PSMA-617 from 0.01 to 100 nM concentrations.

nM	%bound/mg	SD
0.01	76.66	11.42
0.10	62.00	11.30
0.50	17.68	4.54
1.00	10.05	2.65
5.00	3.76	1.16
10.00	3.41	2.82
50.00	0.56	0.22
100.00	0.27	0.10

Cellular uptake and competitive binding:

TABLE S2. The cellular uptake results of 1 nM [^{47}Sc]Sc-PSMA-617 in LNCaP, LNCaP + 100 µM 2-PMPA, PC-3, and PC-3 + 100 µM 2-PMPA after 1 h incubation at 37°C.

LNCaP		LNCaP + 2-PMPA		PC-3		PC-3 + 2-PMPA	
%bound/mg	SD	%bound/mg	SD	%bound/mg	SD	%bound/mg	SD
56.88	6.52	2.34	0.78	1.82	0.43	1.68	0.43

Internalization:

TABLE S3. The rate of internalization of 1 nM of [^{47}Sc]Sc-PSMA-617 up to 72 h.

Time (h)	%Surface bound	SD	%Internalized	SD
0.50	66.02	1.11	33.98	1.11

1.00	62.67	4.69	37.33	4.69
2.00	55.28	0.90	44.72	0.90
4.00	47.08	2.17	52.92	2.17
6.00	41.49	2.80	58.51	2.80
24.00	29.55	3.80	70.45	3.80
48.00	27.12	3.19	72.88	3.19
72.00	28.12	1.07	71.38	1.07

***In vivo* PET studies with [^{43}Sc]Sc-PSMA-617"**

***In vivo* imaging:**

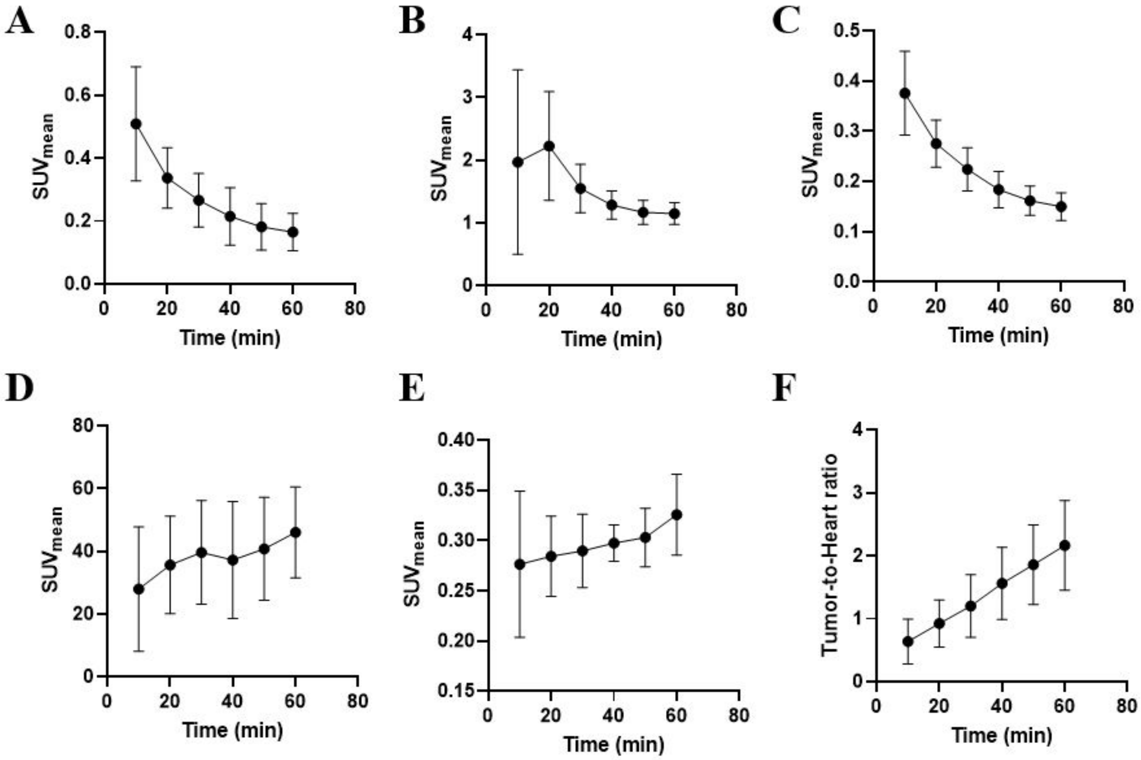

FIGURE S3. Time activity curves of select organs from a 60 min dynamic scan, (A) Heart, (B) Kidney, (C) Liver, (D) Bladder, (E) Tumor, and (F) the Tumor-to-Heart ratio.

TABLE S4. The time activity results, represented as SUV_{mean} values, from the 60 min dynamic scan of [^{43}Sc]Sc-PSMA-617 in mice bearing LNCaP tumors.

**Time Frame (min)**	**Heart**		**Kidney**		**Liver**		**Bladder**		**Tumor**	
	SUV_{mean}	SD	SUV_{mean}	SD	SUV_{mean}	SD	SUV_{mean}	SD	SUV_{mean}	SD
10.00	0.51	0.16	2.23	0.75	0.38	0.07	27.96	17.14	0.28	0.06
20.00	0.34	0.08	1.55	0.34	0.28	0.04	35.66	13.48	0.28	0.03
30.00	0.27	0.07	1.28	0.20	0.22	0.04	39.62	14.31	0.29	0.03
40.00	0.22	0.08	1.17	0.17	0.18	0.03	37.22	16.16	0.30	0.02
50.00	0.18	0.06	1.15	0.15	0.16	0.03	40.78	14.23	0.30	0.03
60.00	0.17	0.05	1.29	0.19	0.15	0.02	46.04	12.52	0.33	0.03

***In vivo* specificity:**

TABLE S5. The complete biodistribution of 1.5 h [^{43}Sc]Sc-PSMA-617 in mice bearing LNCaP tumors, mice bearing PC-3 tumors, and mice bearing LNCaP tumors co-injected with 2-PMPA(blocking).

**Organ**	**1.5 h LNCaP**		**1.5 h Blocking LNCaP**		**1.5 h PC3**	
	%ID/g	SD	%ID/g	SD	%ID/g	SD

Blood	0.23	0.02	0.17	0.03	0.04	0.01
Heart	0.80	0.12	0.35	0.14	0.34	0.21
Lungs	1.21	0.06	0.98	0.67	0.69	0.41
Pancreas	0.57	0.24	0.36	0.12	0.32	0.22
Spleen	0.83	0.49	0.26	0.13	0.84	0.30
Stomach	0.11	0.05	0.44	0.24	0.07	0.02
Liver	0.75	0.14	1.04	1.10	0.88	0.18
Kidney	5.05	1.44	0.62	0.78	3.92	2.23
S. Intestine	0.54	0.05	0.67	0.35	0.17	0.09
L. Intestine	0.97	1.17	0.47	0.26	0.11	0.10
Fat	0.76	0.17	0.72	0.31	0.06	0.01
Skin	0.60	0.10	0.21	0.09	0.48	0.42
Muscle	0.56	0.31	1.16	0.44	0.43	0.38
Femur	0.60	0.04	0.64	0.28	0.42	0.25
Brain	0.12	0.02	0.25	0.12	0.14	0.07
Tumor	2.20	0.26	0.46	0.19	0.28	0.23

Extended *in vivo* imaging:

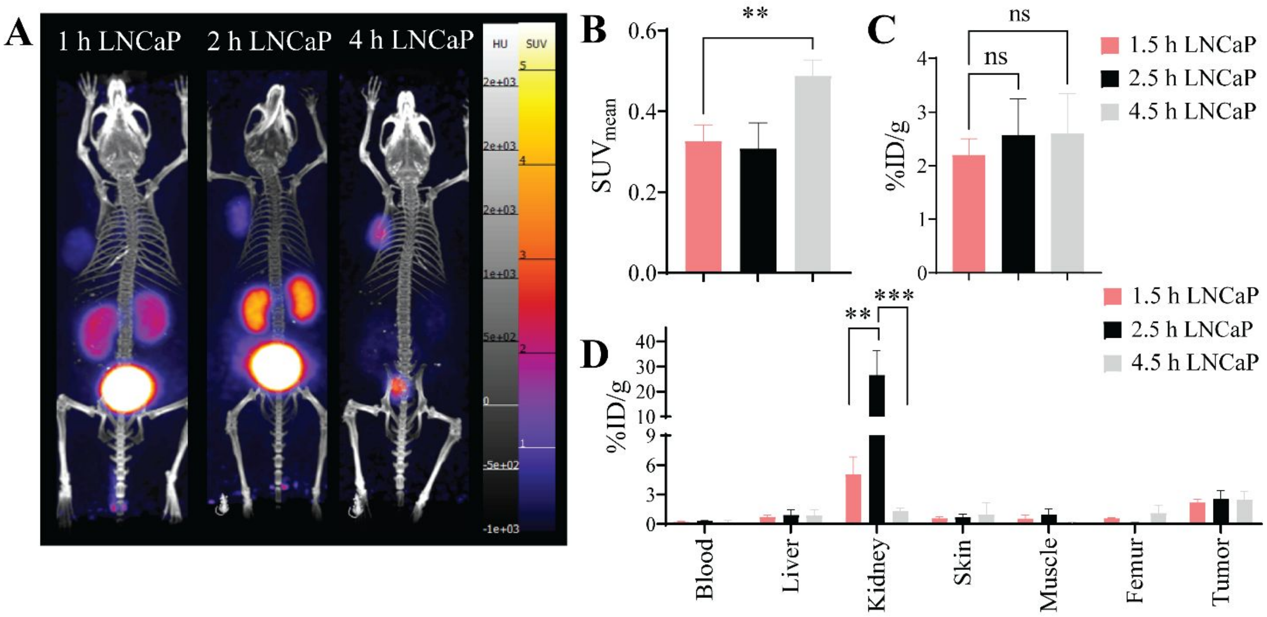

FIGURE S4. (A) MIPs of 30-min [^{43}Sc]Sc-PSMA-617 PET scans of animals bearing LNCaP-tumors at 1, 2, and 4 h post-injection. All images are windowed the same for comparison. (B) PET SUV$_{mean}$ comparison between the 1 h LNCaP of [^{43}Sc]Sc-PSMA-617, 2 h LNCaP of [^{43}Sc]Sc-PSMA-617 and 4 h LNCaP of [^{43}Sc]Sc-PSMA-617, analyzed using a one-way ANOVA. (C) The %ID/g comparison between the 1.5, 2.5, and 4.5 h LNCaP tumors were analyzed using a one-way ANOVA. (D) The biodistribution of the three groups at 1.5, 2.5, and 4.5 h post-injection, represented as %ID/g, where the y-axis is split. The upper y-axis is for better visualization of the kidney comparisons, while the lower y-axis is for improved visualization of the tumor values. All groups were n=4. $*p < 0.05$, $**p < 0.01$, $***p < 0.001$, $****p < 0.0001$

TABLE S6. The complete biodistribution of a 1.5, 2.5 and 4.5 h post injection of [^{43}Sc]Sc-PSMA-617 in mice bearing LNCaP tumors.

	**1.5 h LNCaP**		**2.5 h LNCaP**		**4.5 h LNCaP**	
	%ID/g	SD	%ID/g	SD	%ID/g	SD

Blood	0.23	0.02	0.32	0.01	0.18	0.15
Heart	0.80	0.12	0.50	0.30	0.38	0.25
Lungs	1.21	0.06	0.98	0.41	1.09	0.89
Pancreas	0.57	0.24	0.87	0.07	0.28	0.15
Spleen	0.83	0.49	0.69	0.31	1.43	1.07
Stomach	0.11	0.05	0.35	0.25	0.09	0.04
Liver	0.75	0.14	0.94	0.46	0.85	0.53
Kidney	5.05	1.44	26.66	8.41	1.38	0.28
S. Intestine	0.54	0.05	0.80	0.27	0.32	0.25
L. Intestine	0.97	1.17	0.36	0.14	0.24	0.07
Fat	0.76	0.17	0.54	0.42	1.33	1.02
Skin	0.60	0.10	0.70	0.29	1.00	1.02
Muscle	0.56	0.31	0.98	0.50	0.16	0.06
Femur	0.60	0.04	0.15	0.01	1.12	0.71
Brain	0.12	0.02	0.15	0.01	0.15	0.10
Tumor	2.20	0.26	2.56	0.69	2.46	0.69

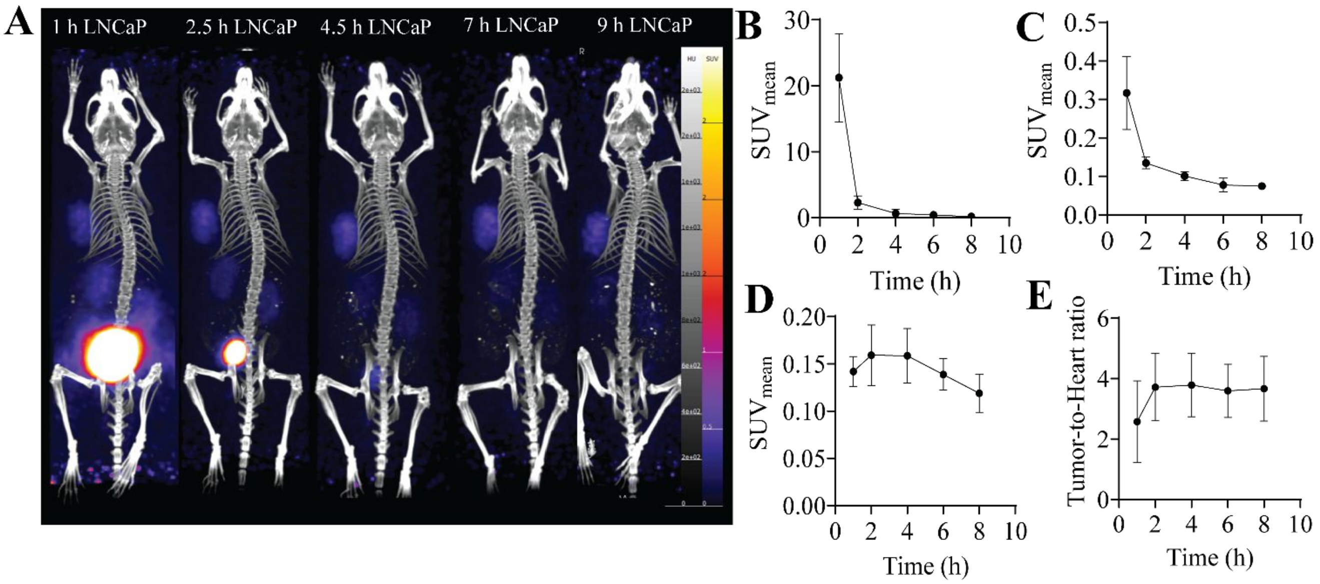

FIGURE S5. MIPs of 30-min [^{43}Sc]Sc-PSMA-617 PET scans of animals bearing LNCaP tumors at 1, 2.5. 4.5, 7, and 9 h post-injection. The time activity curves of select organs: (B) Heart, (C) Kidney, (D) Tumor and (E) tumor-to-heart ratio. All groups were n=4.

TABLE S7. The time activity results of LNCaP bearing mice injected with [^{43}Sc]Sc-PSMA-617 and scanned at 1, 2.5, 4.5, 7, and 9 h.

**Time (h)**	**Brain**		**Heart**		**Lung**		**Liver**	
	SUV_{mean}	SD	SUV_{mean}	SD	SUV_{mean}	SD	SUV_{mean}	SD
1.00	0.02	<0.01	0.07	0.02	0.06	0.01	0.07	0.03
2.50	0.01	<0.01	0.04	0.01	0.04	0.01	0.04	0.01
4.50	0.01	<0.01	0.04	0.01	0.03	0.01	0.03	0.01
7.00	0.01	<0.01	0.04	0.01	0.03	0.01	0.04	0.00
9.00	0.01	<0.01	0.03	0.01	0.03	0.01	0.03	<0.01
**Time (h)**	**Kidney**		**Bladder**		**Muscle**		**Tumor**	

	SUV_{mean}	SD	SUV_{mean}	SD	SUV_{mean}	SD	SUV_{mean}	SD
1.00	0.32	0.08	21.23	5.81	0.06	0.02	0.14	0.01
2.50	0.13	0.01	2.72	0.76	0.01	<0.01	0.16	0.03
4.50	0.08	0.03	0.61	0.55	0.01	<0.01	0.14	0.02
7.00	0.09	0.01	0.38	0.52	0.01	<0.01	0.14	0.01
9.00	0.07	<0.01	0.12	0.06	0.01	<0.01	0.12	0.02

Comparative biodistribution of [^{43}Sc]Sc-PSMA-617 and [^{68}Ga]Ga-PSMA-617:

TABLE S8. The complete biodistribution of a 1.5 h post injection of [^{43}Sc]Sc-PSMA-617 and[^{68}Ga]Ga-PSMA-617 in mice bearing LNCaP tumors.

	**1.5 h [^{43}Sc]Sc-PSMA-617**		**1.5 h [^{68}Ga]Ga-PSMA-617**	
	%ID/g	SD	%ID/g	SD
Blood	0.23	0.02	0.30	0.11
Heart	0.80	0.12	0.39	0.08
Lungs	1.21	0.06	0.98	0.26
Pancreas	0.57	0.24	0.36	0.18
Spleen	0.83	0.49	1.01	0.26
Stomach	0.11	0.05	0.13	0.06
Liver	0.75	0.14	4.95	1.54
Kidney	4.92	1.27	3.25	1.67
S. Intestine	0.54	0.05	0.76	0.36
L. Intestine	0.97	1.17	0.29	0.12

Fat	0.76	0.15	1.31	0.79
Skin	0.60	0.10	0.59	0.28
Muscle	0.56	0.31	1.85	0.32
Femur	0.60	0.04	1.01	0.33
Brain	0.12	0.02	0.06	0.01
Tumor	2.20	0.26	2.50	1.12

***In vivo* SPECT imaging with [^{47}Sc]Sc-PSMA-617**

TABLE S9. The complete biodistribution of [^{47}Sc]Sc-PSMA-617 in mice bearing LNCaP tumors at 24 and 48 h.

	**24 h**		**48 h**	
	%ID/g	SD	%ID/g	SD
Blood	<0.01	<0.01	<0.01	<0.01
Heart	<0.01	<0.01	<0.01	<0.01
Lungs	0.01	0.01	0.01	0.00
Pancreas	<0.01	<0.01	<0.01	<0.01
Spleen	0.01	<0.01	<0.01	<0.01
Stomach	0.07	0.05	0.03	0.01
Liver	0.02	<0.01	0.02	<0.01
Kidney	0.13	0.04	0.12	0.02
S. Intestine	0.03	0.01	0.02	0.01
L. Intestine	0.40	0.27	0.14	0.03

Fat	0.01	0.01	0.02	0.02
Skin	0.01	<0.01	0.01	0.01
Muscle	<0.01	<0.01	0.01	<0.01
Femur	<0.01	<0.01	0.01	<0.01
Brain	0.01	<0.01	0.01	<0.01
Tumor	2.37	0.11	2.13	0.10
Tumor/muscle	1532.50	300.10	1588.80	236.80
Tumor/blood	1452.60	288.00	1506.50	86.20
Tumor/kidney	20.38	7.23	18.27	3.05

Longitudinal imaging and therapy study

LNCaP tumor-bearing mice with tumor volumes between 100-150 mm^3 and weights between 25-30 g were imaged with 0.8 nmol of [^{43}Sc]Sc-PSMA-617 3 d prior to administration of a dose of either: 25.9 MBq [^{47}Sc]Sc-PSMA-617 (10.8 nmol), 11.1 MBq [^{47}Sc]Sc-PSMA-617 (4.6 nmol) or saline (control). Tumor measurements (mm) and animal weight (g) were collected every other day. Mice were euthanized when one of the predefined endpoints were met: loss of 20% weight, tumor volumes at >2500 mm^3, or the tumor became necrotic (skin broken down or ulcerations). The tumor dimension was determined by measuring the tumor's longest (L) and widest (w) diameter and calculated using **EQUATION 1**.

EQUATION 1

$$Tumor\ volume\ (mm^3) = 0.5 \cdot (L \cdot w^2)$$

Weight changes were determined by comparing the daily weight to the starting weight measured day of administration of [^{47}Sc]Sc-PSMA-617. The [^{43}Sc]Sc-PSMA-617 PET SUV_{mean} value of each mouse was plotted against survival time.

TABLE S10. The average tumor volumes (mm^3) per cohort.

	High dose		Low dose		Control	
Day	Average	SD	Average	SD	Average	SD
0.00	131.87	25.06	143.36	23.78	127.29	24.14
2.00	191.81	113.81	178.62	75.00	209.60	102.35
4.00	157.15	56.08	154.26	90.25	279.51	153.75
6.00	196.83	131.84	170.49	96.56	332.01	206.55
8.00	154.98	74.46	243.01	90.92	506.01	199.64
10.00	204.60	100.89	353.82	147.10	780.53	418.06
12.00	240.95	149.19	455.35	215.54	1160.44	325.26
14.00	240.53	217.76	640.16	469.13	1485.56	415.04
16.00	298.86	289.74	528.80	249.31	1344.21	251.39
18.00	206.62	98.40	684.95	343.31	1446.96	77.90
20.00	204.55	83.52	834.24	404.29	1671.78	-
22.00	224.61	88.10	879.78	433.10		
24.00	249.98	84.37	920.64	462.91		
26.00	337.79	137.32	850.83	542.24		
28.00	373.00	152.37	940.42	572.12		
30.00	360.82	170.35	1253.08	518.54		
32.00	387.32	219.29	1705.37	336.06		
34.00	467.83	290.03	1530.49	144.08		
36.00	493.53	327.94	1676.90	204.76		
38.00	553.79	342.22	1784.13	205.04		
40.00	654.88	370.79	2056.90	289.07		
42.00	710.51	372.50				
44.00	772.60	383.56				
46.00	835.70	351.88				
48.00	974.55	550.73				
50.00	1158.34	531.81				
52.00	1328.27	454.38				
54.00	1533.11	607.66				
56.00	1614.57	593.83				
58.00	1710.56	630.48				
60.00	1526.28	426.54				
62.00	1516.78	213.42				

64.00	1643.50	302.75
66.00	1696.96	366.53
68.00	1550.36	-
70.00	1666.70	-

TABLE S11 The average relative weight change of the three cohorts.

	High dose		Low dose		Control	
Day	Average	SD	Average	SD	Average	SD
0.00	1.00	0.00	1.00	0.00	1.00	0.00
2.00	1.00	0.03	0.97	0.14	1.00	0.13
4.00	0.99	0.03	0.96	0.13	0.99	0.10
6.00	0.99	0.03	0.95	0.16	0.97	0.07
8.00	0.99	0.03	0.96	0.13	0.97	0.09
10.00	1.00	0.03	0.97	0.15	0.97	0.07
12.00	0.98	0.02	0.96	0.14	1.02	0.12
14.00	0.93	0.06	0.92	0.11	1.00	0.11
16.00	0.97	0.08	0.93	0.14	0.95	0.07
18.00	0.98	0.08	0.93	0.09	0.98	0.13
20.00	1.01	0.10	0.93	0.09	0.83	-
22.00	1.02	0.10	0.91	0.10		
24.00	1.00	0.08	0.92	0.11		
26.00	0.96	0.08	0.91	0.12		
28.00	0.92	0.08	0.92	0.11		
30.00	0.94	0.05	0.91	0.12		
32.00	0.93	0.07	0.93	0.08		
34.00	0.98	0.08	0.92	0.10		
36.00	0.97	0.07	0.90	0.11		
38.00	0.97	0.09	0.93	0.12		
40.00	0.98	0.07	0.89	0.11		
42.00	1.00	0.04				
44.00	0.94	0.07				
46.00	0.96	0.07				
48.00	0.96	0.06				
50.00	0.95	0.04				
52.00	0.95	0.04				
54.00	0.96	0.04				
56.00	0.95	0.05				
58.00	0.95	0.05				
60.00	0.94	0.04				

62.00	0.96	0.03
64.00	0.99	0.04
66.00	0.98	0.02
68.00	1.04	-
70.00	1.05	-

References

1. Cingoranelli SJ, et al. Production and purification of 43Sc and 47Sc from enriched [46Ti]TiO2 and [50Ti]TiO2 targets. *Scientific Reports.* 2023;13:22683.

2. Cingoranelli SJ, et al. Cross section measurements for the production of 49,51Cr and 47Sc from proton irradiation of natural *Applied Radiation and Isotopes.* 2024.

3. Cingoranelli SJ, et al. Production of high purity 47Sc from proton irradiation of natural vanadium targets. *EJNMMI Radiopharmacy and Chemistry.* 2024;9:89.
